# Cytomegalovirus Latency Fuels Inflammation Following Myocardial Infarction

**DOI:** 10.1161/CIRCRESAHA.125.327799

**Published:** 2026-09-14

**Authors:** Eleni Dapergola, Marko Šustić, DiyaaElDin Ashour, Ebram Tharwat Melika, Maja Cokarić Brdovčak, Jelena Materljan Franki, Mijo Golemac, Berislav Lisnić, Ema Bellulovich, Valérie Boivin-Jahns, Tobias Krammer, Kenz Le Gouge, Lavinia Rech, Verena Stangl, Karl Kashofer, Peter P. Rainer, Roland Jahns, Encarnita Mariotti-Ferrandiz, Hamid Marwan, Maxim Terekhov, Mohammadreza Keshtkar, Antoine-Emmanuel Saliba, Clement Cochain, Panagiota Arampatzi, Mugdha Srivastava, Sven Plein, Yasemin Ekinci, Ioakim Spyridopoulos, Stipan Jonjić, Ulrich Hofmann, Stefan Frantz, Ilija Brizić, Georg Gasteiger, Gustavo Campos Ramos

**Affiliations:** 1 Würzburg Institute of Systems Immunology, Max Planck Research Group at the Julius-Maximilians-University Würzburg, Germany (E.D., G.G.).; 2 Department of Internal Medicine, Faculty of Medicine (M. Šustić), University of Rijeka, Croatia.; 3 Center for Proteomics, Faculty of Medicine (M.C.B., J.M.F., B.L., E.B., S.J., I.B.), University of Rijeka, Croatia.; 4 Department of Histology, Faculty of Medicine (M.G.), University of Rijeka, Croatia.; 5 Department of Internal Medicine I (D.E.D.A., E.T.M., U.H., S.F., G.C.R.), University Hospital Würzburg, Germany.; 6 Comprehensive Heart Failure Center (D.E.D.A., E.T.M., H.M., M.T., M.K., U.H., S.F., G.C.R.), University Hospital Würzburg, Germany.; 7 Interdisciplinary Bank of Biomaterials and Data Würzburg (ibdw) (R.J.), University Hospital Würzburg, Germany.; 8 Institute of Experimental Biomedicine (C.C.), University Hospital Würzburg, Germany.; 9 Institute of Pharmacology and Toxicology (V.B.-J.), University of Würzburg, Germany.; 10 Core Unit Systems Medicine (P.A., M. Srivastava), University of Würzburg, Germany.; 11 Helmholtz Institute for RNA-based Infection Research, Helmholtz Centre for Infection Research, Würzburg, Germany (T.K., A.-E.S.).; 12 Division of Cardiology, Department of Internal Medicine, University Heart Center, Medical University of Graz, Austria (K.L.G., L.R., P.P.R.).; 13 BioTechMed Graz, Austria (K.L.G., L.R., P.P.R.).; 14 Department of Internal Medicine, Landeskrankenhaus Südweststeiermark, Austria (V.S.).; 15 Diagnostic and Research Institute of Pathology, Medical University of Graz, Austria (V.S.).; 16 Diagnostic and Research Institute of Pathology, Diagnostic and Research Center of Molecular BioMedicine, Medical University of Graz (K.K.).; 17 Immunoregulation-Immunopathology-Immunotherapy, UMRS959, Sorbonne Université, INSERM, Paris, France (E.M.-F.).; 18 Institut Universitaire de France, Paris, France (E.M.-F.).; 19 Department of Medicine, St. Johann in Tirol General Hospital, Austria (P.P.R.).; 20 Université Paris Cité, Inserm, PARCC Paris, France (C.C.).; 21 Department of Biochemistry, Faculty of Pharmacy, Minia University, Egypt (E.T.M.).; 22 Leeds Institute of Cardiovascular and Metabolic Medicine, University of Leeds, United Kingdom of Great Britain & Northern Ireland (S.P.).; 23 Newcastle University, Translational Research Institute, Vascular Biology and Medicine Theme, Faculty of Medical Sciences (I.S., Y.E.).; 24 Freeman Hospital, Cardiology, Newcastle-Upon-Tyne, United Kingdom of Great Britain & Northern Ireland (I.S.).

**Keywords:** cytomegalovirus, epitopes, flow cytometry, myocardial infarction, phenotype

## Abstract

**BACKGROUND:**

Epidemiological studies have consistently associated cytomegalovirus (CMV) seropositivity with adverse cardiovascular outcomes. However, the mechanisms by which cytomegalovirus infection impacts pathophysiology in the heart remain poorly understood. In this study, we sought to dissect how latent murine cytomegalovirus infection impacts cardiac immune cell dynamics at steady-state and during postmyocardial infarction (MI) repair.

**METHODS:**

Experimental MI studies were conducted in C57BL/6J mice previously infected with murine cytomegalovirus. In situ inflammatory responses were characterized by spectral flow cytometry, bulk and single-cell RNA/T-cell receptor (TCR) sequencing, whereas cardiac function was monitored by echocardiography and cardiac magnetic resonance imaging. Moreover, we retrospectively assessed the cytomegalovirus serostatus and associated T-cell expansions in a well-characterized patient cohort with longitudinal cardiac magnetic resonance imaging data available and performed bulk TCR sequencing on peripheral blood and myocardial samples to identify cytomegalovirus-specific TCRs.

**RESULTS:**

Our findings show that exposure to murine cytomegalovirus induces long-term changes in the cardiac transcriptional profile and alterations in various cardiac-resident immune cell populations, including the establishment of virus-specific memory CD8^+^ T-cell residency. Compared with infarcted controls, mice previously exposed to murine cytomegalovirus exhibited stronger inflammatory responses marked by increased CD8^+^ T-cell infiltration and worsened cardiac function following MI. These observations in mice were supported by data from patients with cytomegalovirus seropositive MI, who harbored cytomegalovirus-responsive T cells in the heart.

**CONCLUSIONS:**

Our findings demonstrate that latent cytomegalovirus infection leads to long-term changes in the cardiac microenvironment, which ultimately impair post-MI healing outcomes.

**Novelty and Significance**

**What Is Known?**

- Immune responses are required for postmyocardial infarction (MI) repair, but they can also cause collateral damage.
- Latent cytomegalovirus (CMV) infection can profoundly reshape an individual’s immune profile.
- Cytomegalovirus seropositivity is epidemiologically linked to worse cardiovascular outcomes.

**What New Information Does This Article Contribute?**

- Latent murine cytomegalovirus (MCMV) infection persistently remodels the cardiac immunologic landscape and promotes seeding of tissue-resident CD8^+^ T cells in the myocardium.
- Latent MCMV fuels inflammation after myocardial infarction and aggravates adverse ventricular remodeling in mice.
- An exacerbated cytomegalovirus-specific immune response is associated with worse long-term cardiac functional outcomes in seropositive patients.

Immunologic mechanisms impact the quality of post-MI repair and subsequent heart failure development. Individual immune profiles are shaped by natural exposure to common infections, yet how these exposures influence myocardial immune responses and post-MI outcomes remains poorly understood. In the present study, we combined experimental models of MI superimposed with common viral infections, including MCMV, to recapitulate more realistic clinical scenarios. Our study reveals that MCMV infection impacts the cardiac tissue-resident immune cell population, marked by increased proinflammatory gene expression signatures across various cardiac cell populations and by promoting the seeding of virus-specific memory CD8^+^ T cells in the heart. When preexposed to MCMV infection, mice undergoing MI showed increased in situ inflammation and worsened cardiac functional outcomes. Retrospective analysis of independent clinical cohorts confirmed the association between cytomegalovirus infection, exacerbated inflammation, and worsened cardiac functional outcomes. These findings underscore how individual viral exposure and immune profile shape the inflammatory response and outcomes following MI.

**Meet the First Author, see p e000771**

**Editorial, see Article by Cremonesi and Kallikourdis**

Immunologic processes play key roles in a broad range of myocardial diseases, including postmyocardial infarction (MI) repair and heart failure (HF).^1^ Tissue immunology is shaped by individual histories of antigenic encounters, and common viral infections may therefore influence pathophysiological mechanisms in the heart. However, the possible links between antiviral immunity and myocardial diseases remain poorly understood. Among the most common viral infections in humans, cytomegalovirus shows particularly high seroprevalence, exceeding 60% in elderly populations.^2,3^ These observations are significant because latent cytomegalovirus infection is considered a major driver of variation in individual immune profiles.^4^ Moreover, the age-related rise in cytomegalovirus seropositivity parallels the increased incidence of MI and HF in the elderly.^5^ In Germany, for instance, the average age of first MI occurrence is 66.6 years for men and 75.3 years for women, whereas the prevalence of HF increases from <1% in individuals aged 45 to 55 years to over 10% among those in their 80s.^6,7^

Cytomegalovirus is one of the most ubiquitous human herpesviruses and can establish persistent, or even completely latent, infection in its host with occasional virus reactivation.^8,9^ Cytomegalovirus infection elicits a characteristic CD8^+^ T-cell response distinguished by a phenomenon known as memory inflation. In this process, T cells targeting specific viral epitopes gradually expand over time and, in older individuals, can comprise up to 50% of the entire pool of circulating CD8^+^ T cells.^10^ This leads to a markedly skewed TCR (T-cell receptor) repertoire, with the affected cells predominantly exhibiting both terminal differentiation phenotype and cellular senescence features.^11^ Moreover, latent cytomegalovirus infection generates chronic low-level inflammation characterized by increased levels of circulating cytokines, such as IFN (interferon)-γ, TNF (tumor necrosis factor), and IL (interleukin)-6.^5,12^ Not only are these cytokines elevated in patients with chronic HF, but IFN-γ also induces detrimental derangements in cardiomyocyte metabolism as characterized by a decreased oxidative phosphorylation rate,^13,14^ a phenotype commonly associated with some forms of chronic HF.^15^ Four decades ago, Adam et al reported an association between cytomegalovirus infection and atherosclerotic disease, as they observed higher cytomegalovirus IgG antibody levels in patients undergoing vascular surgery compared with matched controls.^16^ Subsequently, several large epidemiological studies have explored the connections between cytomegalovirus serostatus and the incidence and prognosis of cardiovascular disease,^17–20^ and a recent meta-analysis concluded that cytomegalovirus-positive individuals have a higher risk for cardiovascular mortality.^21^ Furthermore, cytomegalovirus was the single viral agent most often found in the cardiac tissue of patients with fulminant myocarditis,^22^ and although it seldom causes the disease itself,^23^ the presence of cytomegalovirus in the cardiac tissue of patients who died from myocarditis indicates its potential contribution to pathophysiological mechanisms in the heart. Taken together, multiple lines of evidence suggest that latent cytomegalovirus infection and the chronic inflammatory environment that it generates might modulate cardiac outcomes in different pathological conditions.

Despite overwhelming clinical evidence for the association of cytomegalovirus infections with myocardial diseases, the putative mechanisms underlying this link remain elusive. In this study, we investigated immunologic processes during MI superimposed with experimental murine models of viral infections that are common in humans, including murine cytomegalovirus (MCMV), to recapitulate real-life clinical scenarios and to mechanistically dissect how viral infections impact myocardial homeostasis and post-MI inflammation. Our findings reveal that exposure to MCMV led to the establishment of virus-specific T cells in cardiac tissue, which persisted long after the acute infection resolved and transitioned into latency. Through a combination of cellular and functional techniques, including spectral flow cytometry, single-cell RNA/TCR sequencing, echocardiography, and cardiac magnetic resonance imaging (cMRI), we found that latent MCMV infection is associated with profound changes in the cardiac tissue-resident immune cell compartment, increased post-MI inflammatory responses, and pronounced adverse cardiac remodeling. Finally, analysis of patient with MI cohorts stratified by cytomegalovirus serostatus and longitudinal cMRI demonstrated that adaptive immune responses to cytomegalovirus correlate with worsened cardiac function. Collectively, these findings provide initial evidence that clonal expansion of antiviral T cells associated with latent cytomegalovirus infection is linked to poorer post-MI healing outcomes, highlighting novel avenues for stratifying patients according to their individual inflammatory burden.

## Methods

### Data Availability

The detailed methods and additional figures are available in the Supplemental Material and the Major Resources Table in the Supplemental Material. Transcriptomic data sets from this study have been archived in NCBI’s Gene Expression Omnibus database (GSE309570 and GSE309706).

Animal procedures were approved by the local authorities (Regierung von Unterfranken and Animal Welfare Committee at the University of Rijeka) and conformed to the guidelines from Directive 2010/63/EU of the European Parliament on the protection of animals used for scientific purposes.

The use of human tissue samples from Graz conforms to legal and institutional requirements and was approved by the ethics committee of the Medical University of Graz (31-288 ex 18/19). The ETiCS study (Etiology, Titre-Course, and Survival) from Würzburg was approved by the ethics commission of the University of Würzburg (186/09) and met the criteria of the Declaration of Helsinki. All patients provided written informed consent. The FRACTalkine inhibition in Acute myocardial infarction trial (FRACTAL) received ethical approval from the National Research Ethics Service Northeast Ethics Committee, complied with the Declaration of Helsinki, was funded by Novokand Pharma AB (previously Kancera AB; Sweden), and the sponsor was The Newcastle on Tyne Hospitals National Health Service (NHS) Foundation Trust (NHS Research Ethics Committee Reference 21/NW/0178).^24^

### Statistical Analysis

All data are depicted as the mean±SEM together with the distribution of individual values for each group. Sample sizes as well as tests used for each statistical analysis are mentioned in the figure legends. Both graphs and statistical analyses were performed using GraphPad Prism version 10.6.1 for Windows (GraphPad Software, Boston, MA, www.graphpad.com) and RStudio. To compare 2 groups with data following a normal distribution, a 2-tailed *t* test was performed. For multiple comparisons including more than 2 groups, an ordinary 1-way ANOVA was performed. For nonparametric or mixed data, a Tukey multiple comparisons test was conducted. A *P* value <0.05 was considered statistically significant.

## Results

### MCMV Infection Persistently Alters the Cardiac Immunologic Landscape

Previous studies conducted both on postmortem human tissue^22^ and experimental mouse models^25^ have detected cytomegalovirus in the hearts of infected mammals. To determine the kinetics of viral replication in the heart, we infected C57BL/6J mice with MCMV and performed a plaque-forming assay on cardiac tissue at different time points (Figure 1A). As shown in Figure 1B, viral titers peaked at day 5 postinfection (p.i.) and subsequently declined, with no in situ replication detected on day 12 p.i. (Figure 1B). Additionally, using immunohistochemistry, we identified clusters of cigar-shaped infected nuclei at day 5 p.i. (Figure 1C). These findings confirm that MCMV can infect murine hearts, yet potential long-term consequences of this infection have not been dissected in detail. Cytomegalovirus usually causes a brief, mild acute infection, characterized by production of infectious viral particles, but maintains lifelong latency, defined as the absence of productive infection despite persistence of viral DNA in the tissue of the host, with periodic reactivation.^27^ To this end, we investigated the extent of transcriptional changes in the hearts of infected animals during the latent phase of infection. We performed bulk RNA sequencing of cardiac tissue from mock, acutely, and latently infected mice. Principal component analysis plots demonstrated clear separation of the experimental conditions, suggesting distinct transcriptional states of the cardiac tissue in uninfected, as well as in acutely and latently infected animals (Figure 1D). Indeed, when comparing gene expression patterns in hearts from latently infected versus noninfected animals, using a stringent cutoff (*P*_adj_<0.01; Benjamini–Hochberg correction), we detected >500 differentially expressed genes (Figure 1E). Subsequent gene ontology over-representation analysis revealed that the latent MCMV infection caused significant changes in the immune landscape within cardiac tissue. A significant proportion of the overrepresented terms with predominantly upregulated genes was related to inflammatory response and included categories such as cell killing, positive regulation of T-cell activation, and cellular response to interferon beta (Figure 1F). Furthermore, closer inspection of deregulated genes revealed that, compared with uninfected animals, latent MCMV infection caused increased expression of not only numerous interferon-related genes, chemokines, and the components of antigen-presenting machinery but also genes associated with cytotoxic T-cell response and CCR2^+^ macrophages, such as *Gzma*, *Gzmb*, *Cd8*, *Cd3*, and *Ccr2* (Figure 1G). Interestingly, over-represented GO categories with predominantly downregulated genes were related to ATP synthesis and oxidative phosphorylation (Figure 1F and 1G).

**Figure 1.**
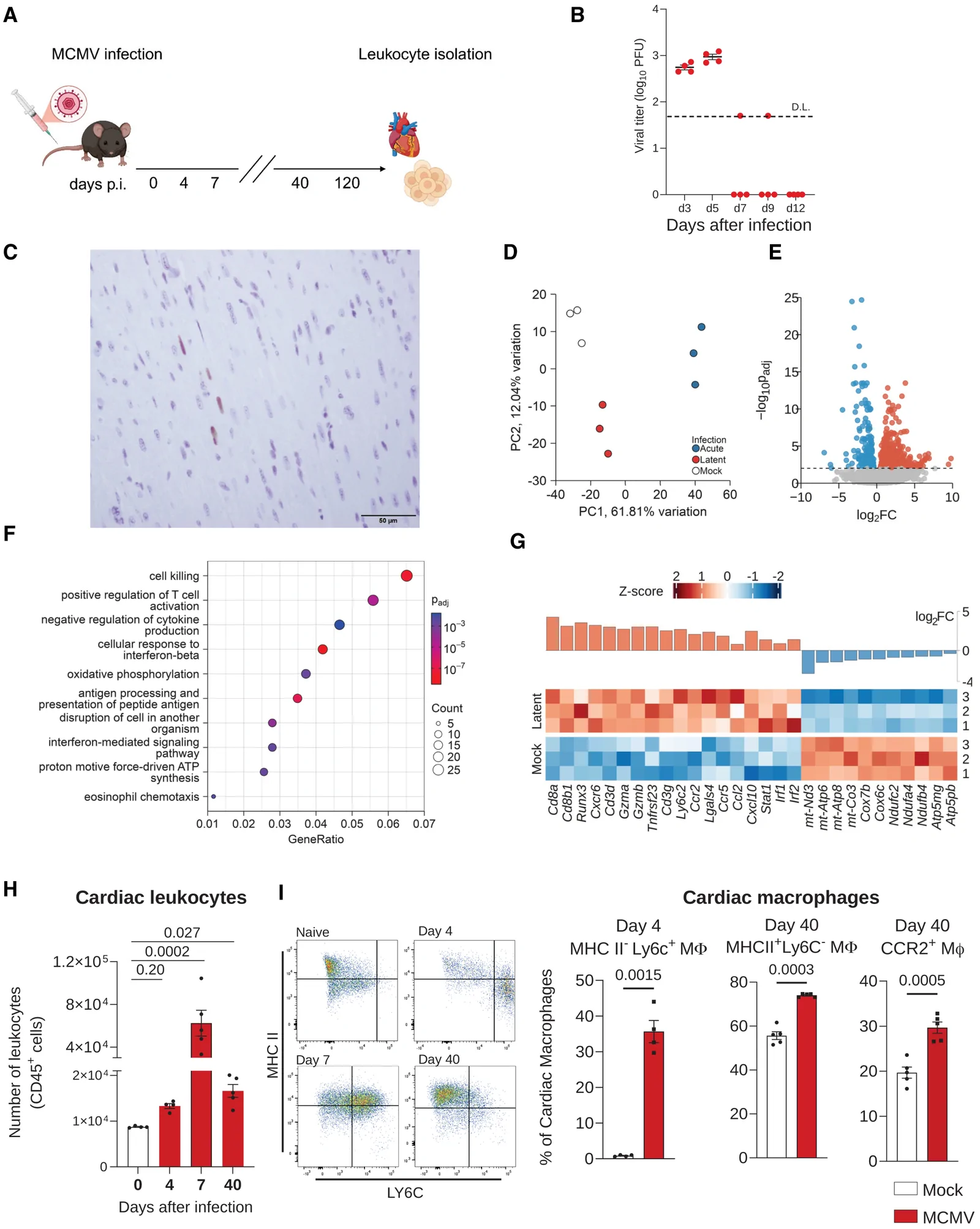
**Cytomegalovirus infection persistently alters the cardiac immunologic landscape. A**, Scheme. Mice were infected intravenously with 2×10^5^ PFU of Δm157 murine cytomegalovirus (MCMV). Noninfected animals were mock-treated with the same volume of sterile PBS. Created in BioRender. Mollar, C. (2026) https://BioRender.com/akkh9s2. **B**, Viral titers, as determined by viral plaque assay, in the hearts of infected mice, at indicated time points (n=4/time point). **C**, Immunohistochemistry staining with MCMV-IE-1 antibody (magnification: 40×). Mice were euthanized at day 5 postinfection. Brown nuclei represent MCMV-infected cells. **D**, PCA plot showing the clustering of RNA transcriptomic data from noninfected, acutely infected (day 5), and latently infected (day 56) mice. **E** through **G**, Differentially expressed genes and over-represented GO terms in latently infected vs noninfected animals. **H**, Leukocyte numbers as determined by flow cytometry in the hearts of MCMV-infected mice at indicated time points after infection. The baseline level (day 0) is set at the number of cardiac leukocytes in noninfected animals. **I**, Representative plots and graphs indicating the phenotypic changes in cardiac monocyte/macrophage populations at indicated time points after infection. Genes with *P*adj <0.01 were considered statistically significantly differentially expressed (**E**, **F**, and **G**). Statistical analysis was performed using an unpaired Student *t* test (**I**) or Kruskal-Wallis test (**H**). Exact *P* values are indicated. Experiments in **H** through **I** were repeated at least 2×. Viral titration (**B**) and RNA sequencing^26^ were each performed once. MHC indicates major histocompatibility complex.

Because the data revealed major changes in cardiac leukocyte composition, we next performed flow cytometry-based phenotyping of cardiac leukocytes (CD45^+^ cells). Leukocyte numbers rose sharply during acute infection, subsequently diminished as the acute phase of the infection ended, but remained ≈2-fold greater during latent infection (day 40) compared with levels in noninfected animals (Figure 1H). Further, cardiac monocyte/macrophage populations underwent phenotypic changes during different phases of MCMV infection. There was an initial influx of Ly6C^+^ MHCII^−^ bona fide monocytes during the first several days of infection. At later time points, the majority of cardiac monocytic cells expressed MHC (major histocompatibility complex class) II molecules and progressively lost Ly6C expression (Figure 1I). Despite these changes in cell dynamics, no overt myocardial necrosis was observed during acute MCMV infection, and no cardiac fibrosis was observed during viral latency (Figure S1). Taken together, these findings demonstrate that MCMV triggers important shifts in the myocardial microenvironment, marked by elevated expression of inflammation-associated genes and persistent shifts in the cardiac-resident immune cell composition in the absence of significant tissue damage.

### MCMV Infection Promotes Seeding of Tissue CD8^+^ T Cells in the Myocardium

Next, we characterized the cardiac CD8^+^ T cells over the course of MCMV infection and determined their antigen specificity and phenotype. The number of cardiac CD8^+^ T cells peaked at day 7 p.i. and remained increased long after acute infection had resolved, even until day 120 p.i. (Figure 2A). Importantly, the majority of cardiac CD8^+^ T cells at day 120 exhibited an antigen-experienced phenotype, as indicated by CD44 expression (Figure 2B). To determine whether these effects were specifically related to MCMV infection, or reflected immune alterations that would be induced more broadly in response to viral infections, we sequentially infected mice with murine gammaherpesvirus 68 (MHV-68), a murine model of the human pathogen Epstein–Barr virus (EBV), followed by MCMV and the WSN strain of Influenza A virus, thereby modeling a series of mild infections that humans typically accumulate from childhood to adulthood^28^ (Figure 2C). Seven months after the last infection, we evaluated the presence of virus-specific CD8^+^ T cells in the hearts and lungs by staining with MHC-I tetramers loaded with peptide antigens of the indicated viruses (pMHC-I tetramer; Figure S2B and S2C). Although influenza- and MHV-68-specific T cells were rarely found in the heart, and to a much lower extent than detected, for example, in the lungs, MCMV infection was associated with an accumulation of antigen-specific CD8^+^ T cells in the heart, suggesting that MCMV infection causes the long-term persistence of virus-specific cells in the heart also in hosts with multiple exposures to infection. To further characterize these cells, we utilized intravascular CD45 staining as well as the expression of CD69, a surrogate marker of tissue residency, and compared the proportions of CD8^+^ T cell subsets in the heart to those in the lung.^29^ Flow cytometry analysis showed that a relatively high fraction of cardiac CD8^+^ T cells was located extravascularly in the tissue (ivCD45^−^) and expressed CD69, as compared with the lung compartment (Figure 2D). We then employed a pMHC-I tetramer specific for the MCMV-derived m139 epitope to identify inflationary antiviral memory CD8^+^ T cells, which expand and persist during MCMV latency.^30^ compared with lungs, a higher proportion of m139^+^ cells in the heart were extravascular, thus indicating the long-term presence of inflationary memory CD8^+^ T cells within cardiac tissue up to 7 months p.i. regardless of any additional exposure to pathogens (Figure 2E). Furthermore, the majority of these ivCD45^−^ cardiac CD8^+^ T cells coexpressed markers consistent with tissue residency, including CXCR6, CD69, CD103, and CD38, and lacked markers of the circulating Teff pool, such as CX3CR1 (C-X3-C motif chemokine receptor 1), Ly6C, and KLRG1 (Killer cell lectin-like receptor G1) (Figure 2F). Taken together, these findings reveal that MCMV infection promotes long-term maintenance of cardiac CD8^+^ T cells expressing surrogate markers typically found among tissue-resident memory T cells.

**Figure 2.**
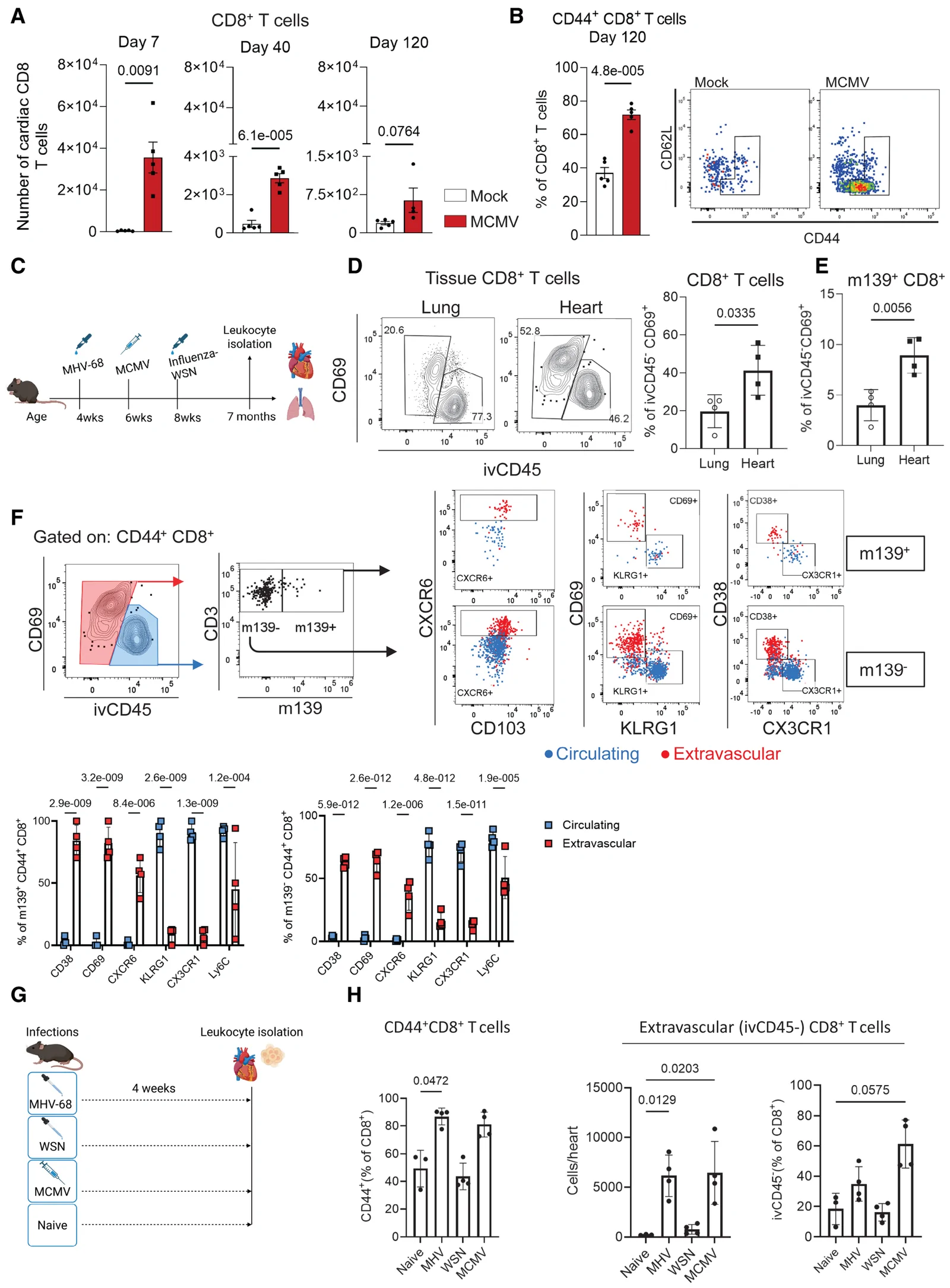
**Cytomegalovirus infection promotes seeding of putative tissue-resident CD8^+^ T cells into the myocardium. A**, Cardiac CD8^+^ T cell numbers at indicated time points. **B**, Percentage of antigen-experienced CD8^+^ T cells in the cardiac tissue at day 120 after murine cytomegalovirus (MCMV) infection. **C**, Scheme. Four-week-old C57BL/6J mice were sequentially infected intranasally with murine gammaherpesvirus 68 (MHV-68), intraperitoneally with MCMV, and intranasally with Wilson-Smith Influenza A (A/WSN). Hearts and lungs were isolated and analyzed 7 months after the last infection **D**. Representative fluorescence-activated cell sorting (FACS) plots displaying the gating of extravascular CD8^+^ T cells in the lung and heart 7 months after sequential infections. **E**, Proportion of extravascular (ivCD45^−^) m139-specific cells in the heart and lung of sequentially infected mice (n=4). **F**, Gating strategy and merged representative FACS plots illustrating the extravascular (ivCD45^−^, shown in red) or circulating (ivCD45^+^, shown in blue) phenotype of m139^+^ (first row on the **right**) and m139^−^ polyclonal (second row on the **right**) CD8^+^ T cells. Their phenotype is characterized by CXCR6, CD69, and CD38 on the *y* axis and CD103, KLRG1 (Killer cell lectin-like receptor G1), and CX3CR1 (C-X3-C motif chemokine receptor 1) on the *x* axis. Quantification panels indicated at the **bottom** (n=4). **G**, Scheme of individual infections. Three groups of 8-week-old C57BL/J6 mice were individually infected intranasally with MHV-68, intraperitoneally with MCMV, and intranasally with influenza A/WSN, and hearts were analyzed 4 weeks post infection. **H**, Graphs showing frequencies of CD44^+^CD8^+^ and absolute cell numbers as well as frequencies of ivCD45^−^ CD8^+^ T cells from the heart of individually infected mice (in naive n=3, in infected n=4). Six to 12-week-old female C57BL/6J mice were used in **A** and **B**, while 8-week-old males were used in **G** and **H**. For **A**, **B**, **D**, and **E**, Statistical analysis was performed using an unpaired Student *t* test or (**G** and **H**) ordinary 1-way ANOVA, and exact *P* values are indicated. Nonsignificant values are not shown. Data representative of 2 experiments. **C** and **G** created in BioRender. Mollar, C. (2026) https://BioRender.com/tqo02x.

To further dissect the relative potential of each of these viral infections to generate cardiac-resident CD8^+^ T cells, mice were individually infected with one of these viruses, and their hearts were analyzed at 4 weeks p.i. (Figure 2G). Interestingly, both MHV-68 and MCMV induced a persistent increase in the numbers of cardiac (ivCD45^−^) CD8^+^ T cells, with MCMV causing the highest fraction of ivCD45^−^ cells (Figure 2H). In contrast, influenza-infected mice showed no increase in absolute numbers of myocardial CD8^+^ T cells. Consistently, both MHV-68 and MCMV infections induced changes in the distribution of cardiac resident macrophages, with MHV-68 promoting a near-complete loss of MHC-II^neg^ macrophage subsets (Figure S3). Although both herpesviruses, MCMV and MHV-68, induced substantial changes in myocardial immune cell populations, the findings related to MCMV infection may be of greater clinical relevance because of its distinctive epidemiology in older adults. EBV seroprevalence reaches about 95% at age 35 and >98% in the elderly, while cytomegalovirus seroprevalence increases continuously with age and could, therefore, serve as a stratifying factor for the elderly. In addition, epidemiological data associate cytomegalovirus infection with a higher risk of cardiovascular death.^21^ Therefore, we sought to assess whether latent cytomegalovirus infection could impact post-MI outcomes.

### Latent MCMV Drives Post-MI Adverse Ventricular Remodeling in Mice

Our preliminary data demonstrated that MCMV-infected animals have an enhanced proinflammatory transcriptional profile correlating with increased leukocyte infiltration. Therefore, to assess the impact of MCMV infection on cardiac remodeling and function in the context of MI, we subjected latently infected animals and noninfected controls to left anterior descending coronary artery ligation (28 days post MCMV infection). Cardiac function was assessed by echocardiography during the acute phase of MI, as well as cMRI on day 56 (Figure 3A). Tissue immunophenotyping was performed on day 5 post-MI. Although no differences in overall survival were observed (Figure 3B), infarcted mice previously infected with MCMV showed overall worsened cardiac function. Echocardiography revealed increased left ventricular end-systolic and end-diastolic diameters in the MCMV+MI group, followed by a reduced fractional shortening at the mid-ventricle level (ie, infarct border zone) compared with MI controls, while cMRI showed reduced ejection fraction and elevated end-systolic and end-diastolic volumes (Figure 3C and 3D).

**Figure 3.**
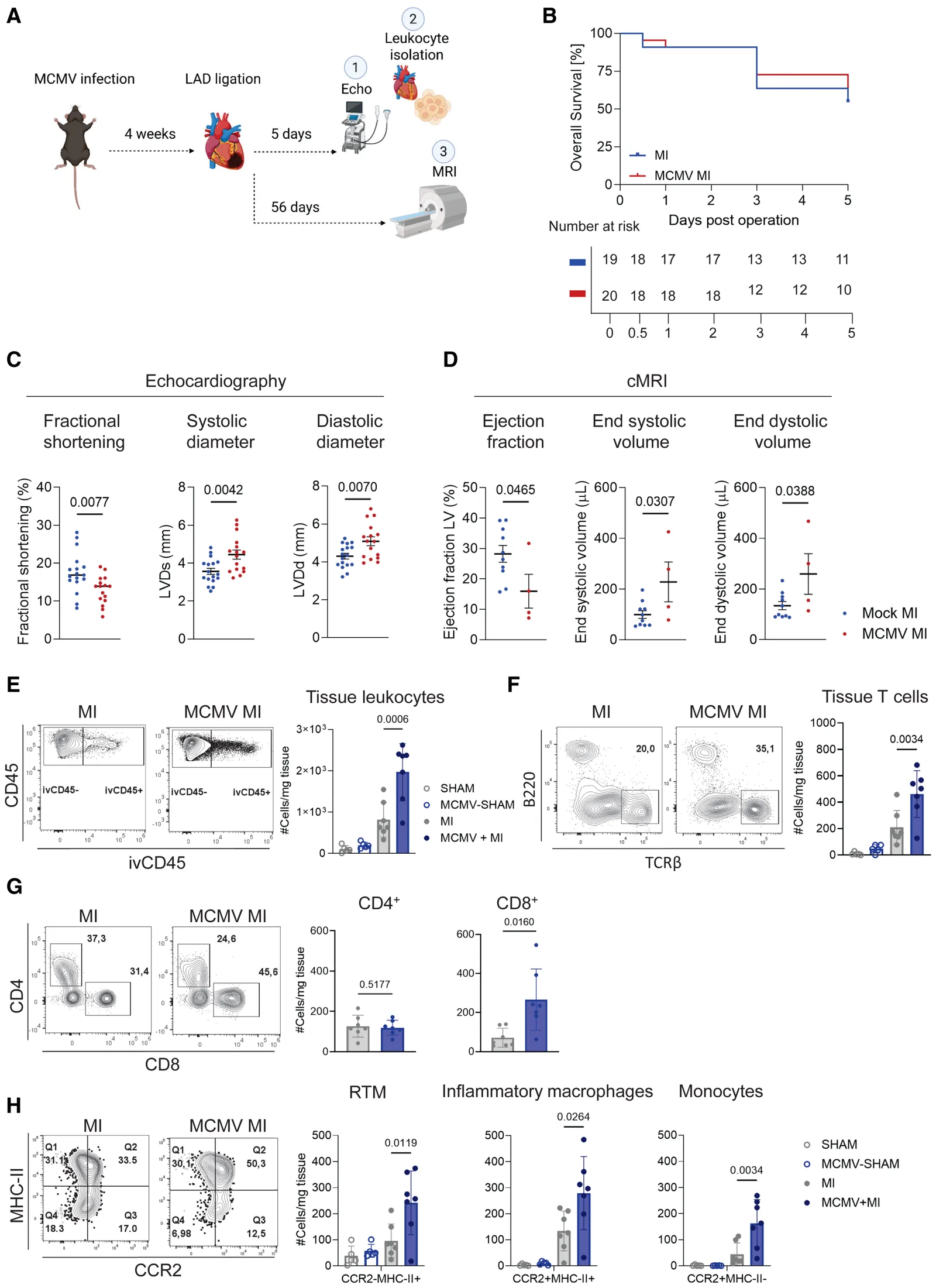
**Murine cytomegalovirus (MCMV) latent infection promotes increased infiltration of inflammatory cells in the infarcted heart. A**, Scheme. Mice were infected with MCMV at 8 weeks of age and underwent LAD ligation at 12 weeks. Analysis was performed 5 days later by echocardiography and cardiac leukocyte phenotyping, or 56 days later by cardiac magnetic resonance imaging (cMRI). Created in BioRender. Mollar, C. (2026) https://BioRender.com/wbikjuf. **B**, Survival after myocardial infarction (MI) with numbers at risk. **C**, Fractional shortening of the left ventricle at the mid-papillary level, just above the akinetic area induced by MI. Diastolic and systolic inner diameters of the left ventricle as measured by echocardiography. In MI, n=17, and in MCMV MI, n=16 **D**. Ejection fraction, end-diastolic volume, and end-systolic volume as determined by cMRI at day 56 after MI. In MI, n=10, and in MCMV MI, n=4. **E**, Total cell numbers/mg in heart tissue and representative FACS plots of total tissue leukocytes and **F**. T cells as well as **G**. separately CD4^+^ and CD8^+^ cells in SHAM (blank gray), MCMV+SHAM (blank blue), MI (full gray), and MI+MCMV (full blue) mice cohorts 5 days post MI. **H**, Total cell numbers/mg of RTMs, inflammatory macrophages, and monocytes in heart tissue 5 days post MI. **E**, **F**, and **H**, Statistics were calculated using 2-way ANOVA and Student *t* test; for SHAM n=5, for both MI groups n=7. **C**, **D**, and **G**, Statistics were calculated using Student *t* test; *P* value is numerically indicated.

Since MCMV infection generates a new steady-state condition in latently infected animals, characterized by chronic inflammatory changes in the pool of heart-infiltrating immune cells of both lymphoid and myeloid lineages, we conducted a detailed characterization of leukocyte populations infiltrating the infarcted heart. Cardiac tissue-resident leukocyte populations, particularly T cells, were elevated in latently infected and infarcted mice (MCMV+MI) compared with infarcted naive controls (Figure 3E and 3F). Notably, this increase was primarily driven by an increase in CD8^+^ T cell numbers in the MCMV+MI group (Figure 3G). Analysis of infiltrating myeloid populations revealed more abundant tissue resident macrophages, inflammatory macrophages, and monocytes in the latently infected infarcted group, as defined by differential expression of CCR2 and MHC-II markers (Figure 3H). Collectively, these results suggest that a previous/latent MCMV infection exacerbates leukocyte infiltration, predominantly involving CD8^+^ T cells, monocytes and macrophages, into the cardiac tissue during the acute phase post MI.

### Prior MCMV Exposure Fuels a Proinflammatory Transcriptional Profile in the Cardiac Tissue of Infarcted Mice

To gain deeper insight into how prior infections shape the transcriptomic response to MI, we performed single-cell RNA sequencing on infarcted hearts from naïve and MCMV-infected mice. After quality control, demultiplexing, and integration of 3 independent experiments, a total of 14 094 leukocytes and 12 844 nonhematopoietic cells were recovered for analysis (Figure S4A through S4C). These included diverse myeloid populations, B cells, and T cells, as well as nonimmune cell types such as endothelial cells, fibroblasts, and epithelial cells. The scRNA-seq data set from Forte et al^31^ was used as a reference for annotating the different cardiac cellular subsets post-MI (Figure 4B; Figure S5B and S5C). Our data revealed a higher fraction of B (*Cd79a*) and T (*Cd3e, Trac*) cells in the previously infected mice and a greater proportion of cells composing the epicardium (*Wt1, Dmkn*) in the MCMV+MI group, compared with naive mice (Figure 4C). As previously shown, these epicardial cells make up the mesothelium covering the heart and are the major source of cardiac fibroblasts during homeostasis.^31^ Several cell types, including cardiac macrophages and myofibroblasts, exhibited an overall proinflammatory state that was absent in the naive MI group (Figure 4D; Figure S6). Gene set enrichment analysis identified a significant upregulation of genes associated with TNF signaling, KRAS signaling, allograft rejection, and inflammatory responses in cardiac macrophages and in *Arg1*-expressing monocytes (Figure S6) of MCMV-exposed mice. Among the upregulated genes were *Il1b*, *Il6*, *Ccl2*, *Cxcl2*, *Cxcr4*, and *Igf1*, reflecting activation of proinflammatory and chemotactic pathways as well as signaling processes associated with immune regulation and tissue myofibroblasts from MCMV+MI mice displayed a similar pattern, with additional responses to interferon-α and -γ, thus indicating involvement in antiviral defense and inflammatory processes.

**Figure 4.**
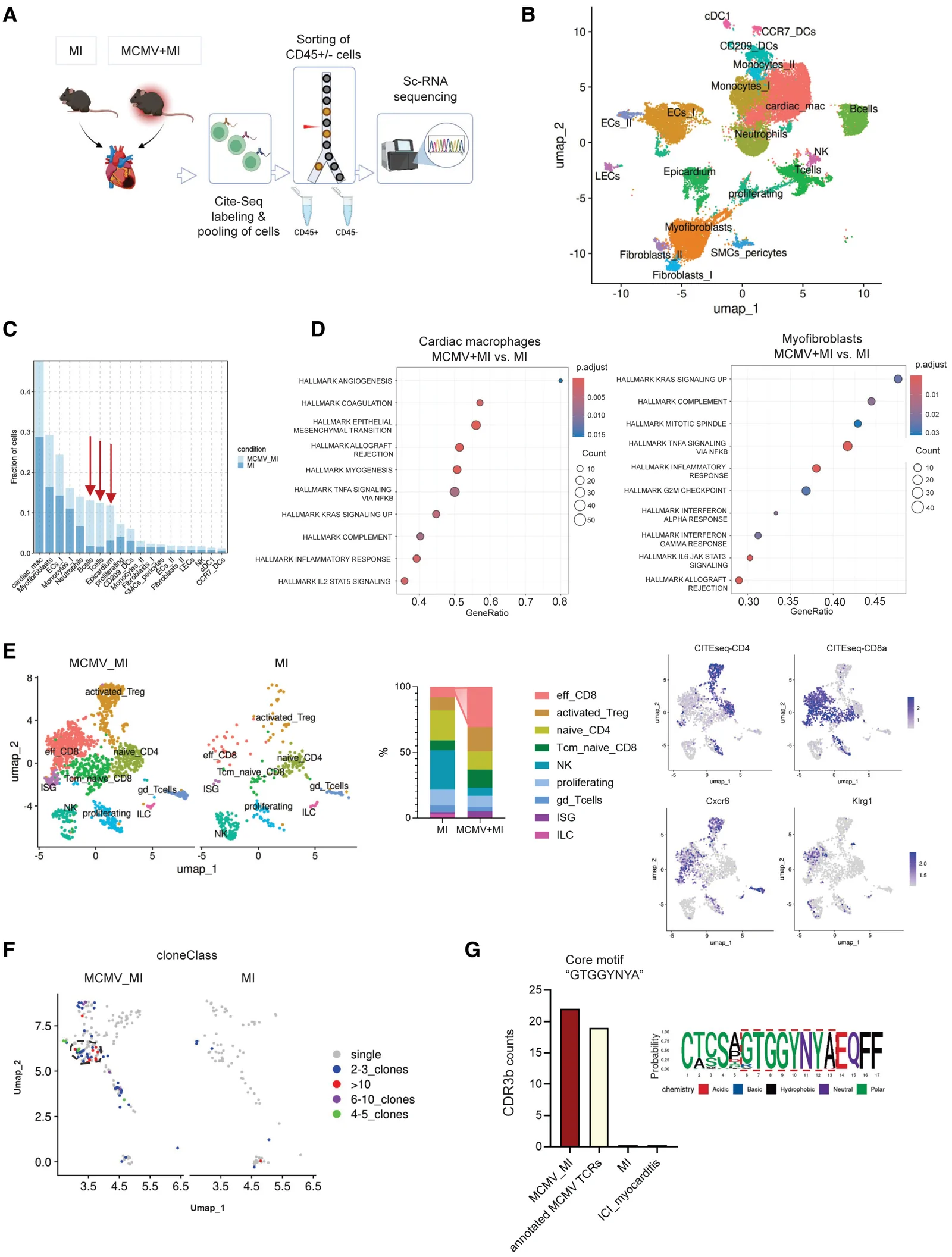
**Prior murine cytomegalovirus (MCMV) exposure induces an elevated proinflammatory transcriptional profile in the cardiac tissue of infarcted mice. A**, Experimental outline for the sc-sequencing pipeline. Infarcted hearts from naive (MI) or MCMV-infected (MCMV+ myocardial infarction [MI]) mice were collected 5 days after MI and processed to obtain a single-cell suspension. Individual mice were labeled with CITE-seq antibodies, and cells were eventually pooled to sort for CD45^+^ and CD45^−^ CD31^+^ and CD45^−^ CD31^−^ cells, and single-cell sequencing was performed using the 10× Genomics platform. Created in BioRender. Mollar, C. (2026) https://BioRender.com/j2zlqmm. **B**, UMAP plot representation of the sorted CD45^+^ and CD45^−^ cells (≈15 000 and 13 000 cells, respectively) from 12-week-old male naive and MCMV-infected mice, 5 days post-MI. **C**, Bar graph representation of the cell proportion of MCMV+MI or MI in each annotated cell cluster. **D**, Gene set enrichment analysis of the top upregulated genes from cardiac macrophages and myofibroblasts in MCMV+MI mice compared with naive controls (MI). The gene list was tested against the Hallmark gene sets from the Molecular Signatures Database (MSigDB). Dot size indicates the number of ranked-list genes that overlap with a gene set. Color denotes the adjusted *P* value from the enrichment test. **E**, UMAP visualization of the subclustered T- and NK-cell subsets for each one of the 2 conditions, and bar graph quantification of the cell proportions. Feature plots indicate CITE-seq expression for CD4 and CD8, as well as *Cxcr6* and *Klrg1* gene expression. **F**, Feature plots of TCR (T-cell receptor) CDR3 sequences indicating clonal expansion in MCMV+MI or MI conditions overlaid on the subclustered CD4 and CD8^+^ T cells that have a productive TCR sequence. Gray indicates a unique TCR CDR3 sequence (single), blue indicates a CDR3 sequence overlapping in 2 to 3 T cells (2–3 clones), green (4–5 clones), violet (6–10 clones), and red (>10 clones) **G**. Bar plot of the number of CDR3 sequences that have an overlapping core motif GTGGYNYA with annotated MCMV-specific TCRs from the Manually Curated Catalogue of Pathology-Associated T Cell Receptor Sequences (McPAS-TCR) database.^32^ And a sequence logo representing the aligned CDR3β amino acid sequences showing conserved positions and variability within the motif.

Lymphocytes are key players in antiviral immunity, and our previous data demonstrated long-lasting reconfiguration and expansion of the cardiac lymphocyte pool after MCMV infection (Figures 1 and 2). Therefore, to globally characterize these changes, we analyzed cardiac lymphocytes based on their transcriptional profile and surface marker expression using genetically barcoded CITE-seq antibodies. These analyses identified activated Foxp3^+^ regulatory T cells (expressing *Icos*, *Ctla4*), effector CD8^+^s (expressing *Cd8a*, *Klrg1*, *Cxcr6*), naive CD4^+^s (*Igfbp4, Ccr7*), central memory and naive CD8^+^s (*Ly6c2*, *Sidt1*), natural killer (NK) cells (*Ncr1, Klra8, Klrb1c*), a cluster of proliferating cells (*Mki67*), γδ T cells (*Trdc*, *Trg-C1*), a cluster with high levels of interferon-stimulated genes (ISG, *Ifit1*, *Isg15*), and group 2 innate lymphoid cells (ILC2, *Gata3*, *Rora*; Figure 4E). Comparison of cell subset distributions between the 2 groups revealed a strong expansion of myocardial effector_CD8 and activated regulatory T cells in the MCMV+MI group. A compiled module score of tissue residency markers (*Cxcr6, Cd69, Cd38*, and *Itgae*, which we termed tissue resident memory [Trm] module score) showed enrichment of this gene signature in effector_CD8, activated regulatory T, and ISG cell subsets (Figure S7A), which corroborated our finding from flow cytometry observed in Figure 2F. Together these findings reveal an activated CD8^+^ T-cell compartment in the post-MI heart of latently infected mice. Overall, these shifts suggest a post-MI response enriched with effector CD8^+^ T cells associated with previous viral exposure.

The scRNAseq approach was further leveraged to monitor the antigen receptors expressed by cardiac T cells, with the purpose of inferring their specificities. After cytomegalovirus infection, virus-specific T cells undergo large clonal expansions during memory inflation, which can be detected by scTCRseq approaches. Consistently, we identified a subset of clonally expanded T cells in the infarcted hearts of MCMV-infected mice but not the naive ones (Figure 4F). Moreover, to infer the antigen specificity of these expanded TCRs, we probed for their sequence similarity with TCRs mapped to MCMV responses in previous studies and curated within the Manually Curated Catalogue of Pathology-Associated T Cell Receptor Sequences (McPAS-TCR) database.^32^ Additionally, we observed that T cells with the curated MCMV-TCR signature were more enriched for the compiled Trm module score and were mainly of the effector_CD8 phenotype (Figure S7B). These findings match our observations from the MCMV-tetramer^+^ analysis (Figure 2F). Next, we applied the GLIPH2 (Grouping of Lymphocyte Interactions by Paratope Hotspots) algorithm,^33^ which models TCR antigen specificity based on sequence similarity of the CDR3 (complementarity-determining region 3). GLIPH2 is critical for determining antigen specificity because it identifies conserved core motifs of short amino acid sequences within the CDR3 region of the TCR that directly contacts the antigenic peptide presented by the MHC molecule. To control for random motif enrichment, the experimental TCR data set is compared against a reference repertoire of 78 116 non-disease-associated mouse TCRs.^33,34^ Using this approach, we described a core CDR3 motif, GTGGYNYA, that exclusively overlapped between the cardiac-expanded TCRs from MCMV-infected mice and the MCMV-specific TCRs annotated in previous studies.^32^ This core TCR motif was not found in noninfected mice, nor in TCRs related to cardiac antigens^35^ (Figure 4G; Figure S7C), suggesting the expansion of MCMV-specific T cells that harbor an enriched tissue residency gene signature in the infarcted myocardium of MCMV-infected mice.

Considering the observed shifts in putative cardiac-resident, virus-specific CD8^+^T cells in MCMV+MI mice, we sought to assess potential causal links between MCMV-induced cardiac CD8^+^ T-cell accumulation and the adverse post-MI remodeling we observed. To selectively deplete CD8^+^ T cells, we treated latently MCMV-infected CD8^iDTR^ mice with diphtheria toxin before and post- MI (Figure S8A). Despite a successful CD8^+^ ablation that was confirmed both in the heart and spleen (Figure S8B through S8D), no significant changes in the immune cell composition or cardiac function were observed. Notably, bulk RNA sequencing of cardiac samples from both groups demonstrated significant enrichment of interferon-response signatures, collagen biosynthesis pathways, and gene sets linked to systolic HF after CD8^+^ depletion (Figure S8E and S8F). Importantly, pathways associated with antiviral defense and cellular responses to viral infection were upregulated in the diphtheria toxin group, suggesting possible viral reactivation following CD8^+^ depletion in infarcted mice (Figure S8F). The enrichment of a transcriptional signature associated with both antiviral responses and systolic HF underscores the challenges and caveats of depleting specific immune cell subsets in mice with MI, particularly in the context of viral infection.

### Presence of Cytomegalovirus-Responsive T Cells in the Cardiac Tissue of MI Patients Links Worse Healing After MI With Cytomegalovirus IgG Titers

Latent cytomegalovirus infection has been increasingly linked to adverse cardiovascular outcomes in patients, with studies showing an association between cytomegalovirus seropositivity and a higher prevalence of coronary heart disease^9^ as well as age-related pathologies including cardiovascular aging.^18^ Our findings from mouse models revealed that cytomegalovirus infection before a cardiac ischemic event resulted in the accumulation of cytomegalovirus-reactive T cells with a phenotype consistent with cardiac residency, an altered inflammatory landscape during the post-MI response, and impaired cardiac healing. To interrogate the translational relevance of these observations, we retrospectively analyzed data available from 2 clinical studies.^24,36^ The study ETiCS includes patients recruited in Würzburg with first acute ST-elevation myocardial infarction (STEMI), including longitudinal cMRI, blood sampling,^36^ and a previously published TCR-seq data set.^37^ Following an approach described in our previous study,^37^ patients from the ETiCS cohort who presented with a reduced left ventricular ejection fraction (LVEF) assessed by cMRI on day 4 post-MI (LVEF <50%) were stratified as good healers or poor healers based on the change in LVEF between index hospitalization and the 12-month follow-up. Good healers were defined as patients with a change in LVEF improvement >13%, whereas poor healers exhibited a change in LVEF improvement <13% (Figure 5A).^37^ This change in LVEF cutoff was defined based on a previous study by Bulluck et al,^38^ which demonstrated that a 13% relative change represents the minimum clinically meaningful improvement in LVEF following acute MI. Moreover, as previously described, after applying this stratification criteria, both good and poor healers showed similar clinical parameters at baseline; such as, age distribution (good healers: 58.0 [±22.0], poor healers: 55.0 [±18.0]; *P*=0.735), sex distribution (good healers: 100%, poor healers: 92%; *P*=0.538), body mass index (good healers: 25.9 [±2.5], poor healers: 28.1 [±4.2]; *P*=0.14), comorbidities such as diabetes (good healers: 23%, poor healers: 24%), and hypertension (good healers: 31%, poor healers: 48%; *P*=0.49), and relative infarct size by late gadolinium enhancement (LGE; good healers: 26 [±9.0], poor healers: 20.5 [±11.3]; *P*=0.149).^37^

**Figure 5.**
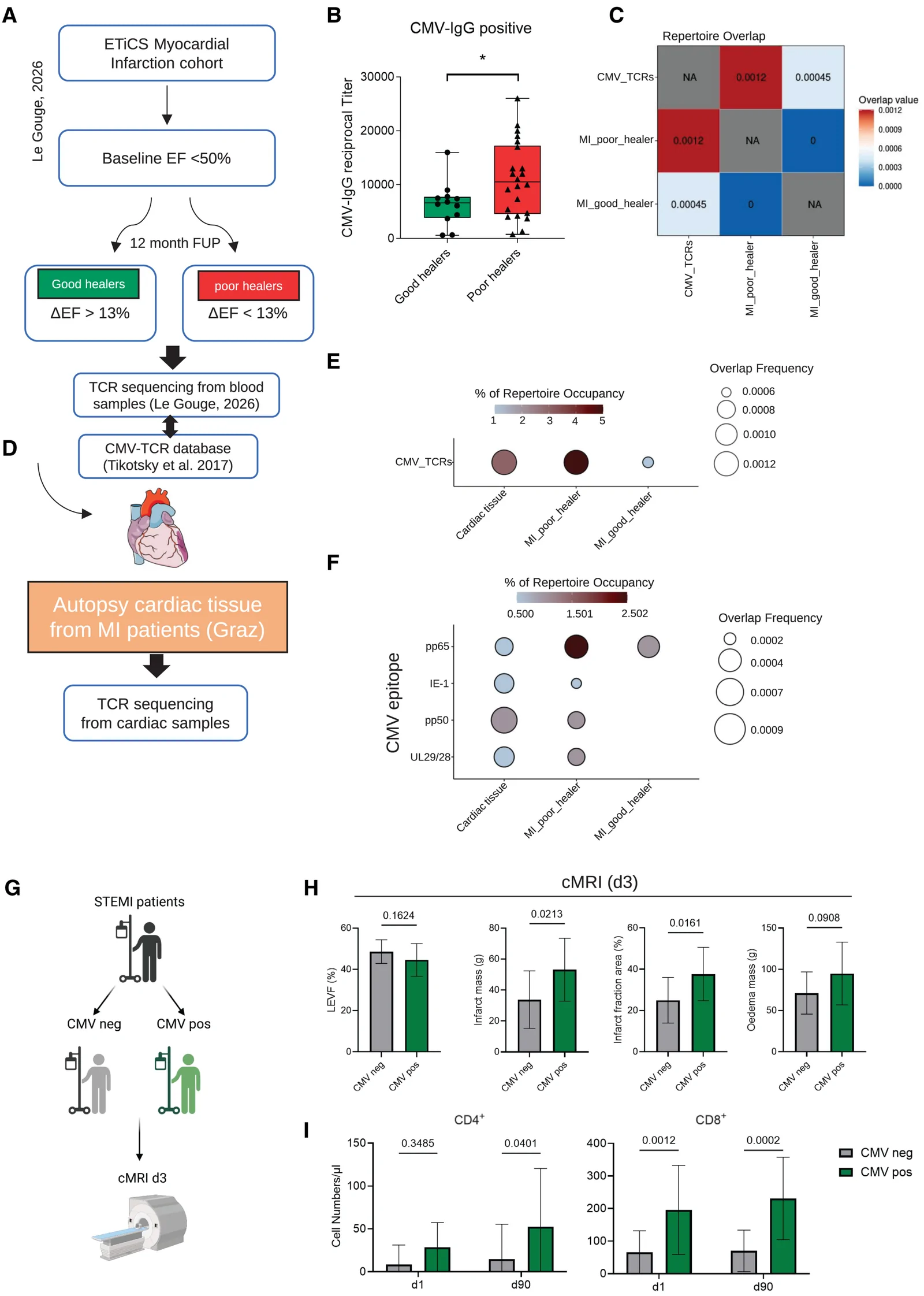
**Presence of cytomegalovirus (CMV)-responsive T cells in the cardiac tissue of patients with myocardial infarction (MI) links worse healing after MI with CMV IgG titers. A**, Study design and change in left ventricular ejection fraction (ΔLVEF)-based stratification of patients with MI from the ETiCS (Etiology, Titre-Course, and Survival) cohort into good or poor healers. **B**, CMV-IgG reciprocal titers from the seropositive patients with MI categorized as good or poor healers. **C**, Heatmap of Jaccard similarity index based on exact CDR3b sequence matches between CMV-annotated T-cell receptors (TCRs)^32^ and TCR repertoires from blood samples of poor or good post-MI healing groups. Color intensity reflects the degree of clonal similarity, with red indicating higher overlap and blue indicating no overlap. **D**, TCR sequencing from cardiac autopsies of acute MI samples and integration with TCRs from blood samples of the ETiCs cohort. **E**, Dotplots of Jaccard similarity index between CMV-annotated TCRs, TCR repertoires from blood samples of poor or good healers, and cardiac autopsies. **F**, Dotplot of the Jaccard similarity index between the same groups, comparing their specificity to defined CMV epitopes. Dot size reflects the degree of clonal similarity, and color intensity indicates the degree of clonal expansion of CMV TCRs within each analyzed group. **G**, Retrospective analysis of the placebo group of the FRACTalkine inhibition in Acute myocardial infarction trial (FRACTAL) trial.^24^ Patients were stratified regarding their CMV serostatus and cMRI was performed 3 days post-ST-elevation myocardial infarction (STEMI) and reperfusion. Created in BioRender. Mollar, C. (2026) https://BioRender.com/43g56vz. **H**, cMRI parameters comparing CMV-negative and positive STEMI patients 3 days post STEMI, reperfusion. **I**, Flow cytometric quantification of blood leukocytes in placebo-treated patients at days 1 and 90 post STEMI. CD4^+^ and CD8^+^ cells are defined as CX3CR1^+^CD28^−^. At day 1 n=12 for CMVpos and n=18 for CMVneg, while at day 90 n=10 for CMVpos and n=18 for CMVneg. **B** and **H**, Statistics were calculated using unpaired Student *t* test; *P* value is numerically indicated. **I**, Statistics were performed using 2way ANOVA and Šídák multiple comparisons test.

Serology analysis in these 2 patient groups revealed that, while good and poor healers had similar cytomegalovirus seropositivity rates, poor healers exhibited higher plasma concentrations of cytomegalovirus-specific IgGs (Figure 5B). To further explore a possible link between cytomegalovirus-specific responses and post-MI outcomes in this cohort, we made use of publicly available TCR sequencing analyses of blood samples from good and poor healers^37^ and computed the overlaps between highly expanded TCRs in good or poor healing patients and human cytomegalovirus-responding TCRs annotated in the McPAS-TCR database.^32^ By computing the Jaccard similarity score between cytomegalovirus-expanded TCRs and those expanded in good or poor healers, which is an index reflecting the proportion of shared TCR sequences relative to the total number of unique sequences, we found that cytomegalovirus-expanded TCRs exhibited more similarity with the poor healer group, which is indicated as a red color on the heatmap (Figure 5C). In addition to cytomegalovirus, we also curated EBV-annotated TCRs from the McPAS-TCR database. We detected EBV-responding TCRs in the donor blood samples, a result we expected given the high prevalence of EBV in the general population.^39^ However, in contrast to cytomegalovirus, no preferential expansion of EBV-annotated TCRs was observed in the poor healer group (Figure S9). Next, we performed TCR sequencing on cardiac tissue obtained from autopsies of patients with deceased MI and biobanked at the Medical University of Graz (Figure 5D). Again, cytomegalovirus-specific TCRs annotated in the McPAS-TCR database^32^ showed overlapped with expanded TCRs found in cardiac samples, suggesting the presence of cytomegalovirus-specific T cells in infarcted hearts. Interestingly, a higher clonal overlap of cytomegalovirus TCRs was observed between cardiac tissue and blood samples from poor healers compared with good healers (Figure 5E). Additionally, TCRs reactive to defined cytomegalovirus epitopes exhibited a distinct overlapping pattern in TCRs from poor healers and those from cardiac tissue. TCRs reactive to the cytomegalovirus epitopes pp50, IE-1, and UL29/28 showed clonal overlap among the TCR repertoire of cardiac samples and blood samples from the poor healers group, but not the good healers group (Figure 5F). By similarly analyzing TCRs reactive to defined EBV epitopes, we noted a distinct pattern of epitope-specific TCRs in the blood samples between the good and poor healer groups. However, cardiac tissue samples had less expansion of EBV epitope-specific TCRs, and there was minimal overlap between EBV-specific TCRs in cardiac tissue and those found in the blood of poor healers (Figure S9). These findings establish a clear association between the presence of cytomegalovirus-reactive T cells in the heart and outcomes after MI, a connection corroborated by our results from experimental mouse models.

An additional retrospective analysis of the placebo cohort from the phase IIa FRACTAL trial^24^ (EudraCT 2021-001354-53) was conducted, in which patients with STEMI were stratified by cytomegalovirus serostatus. Cardiac MRI conducted 3 days after primary percutaneous coronary intervention revealed comparable LVEF between cytomegalovirus-seropositive and -seronegative patients, but a larger infarct size in the cytomegalovirus-seropositive group, measured both as infarct mass and percentage of late gadolinium enhancement (Figure 5G and 5H; Table S2). Analysis of blood leukocyte numbers revealed increased frequencies of terminally differentiated CX3CR1^+^CD28^−^ effector memory CD8^+^ and CD4^+^ T cells in cytomegalovirus-seropositive patients (Figure 5I). Moreover, the blood distribution of CX3CR1^+^CD28^−^ CD8^+^T cells was significantly associated with the infarct sizes (Table S3). These observations mirrored our findings in mice, where the expansion of CX3CR1^+^ intravascular T cells, which is, characteristic of latent cytomegalovirus infection, was associated with the presence of cytomegalovirus-reactive T cells in the heart (Figure 2F), and worse outcome from MI.

These findings in patients with STEMI are consistent with our experimental data from both murine models and human cohorts, further supporting a direct link between cytomegalovirus-related immune changes in the myocardium and adverse remodeling following MI. Our findings have important clinical implications as they suggest that, beyond cytomegalovirus-seropositivity, the expansion of cytomegalovirus-specific T cells could offer relevant information for the stratification of MI patients. Moreover, the potential link of an altered tissue-resident immune compartment in the heart with MI outcome highlights new avenues for further mechanistic studies and the development of innovative immune-targeted therapies.

## Discussion

In this study, we explored how infection with cytomegalovirus, a herpesvirus with high prevalence in elderly patients, affects cardiac homeostasis both in steady-state and following acute MI. In latently infected mice, we observed a notable enrichment of cardiac leukocytes, particularly cardiac T cells expressing surrogate markers of tissue residency and monocyte-derived macrophages, accompanied by a distinct proinflammatory transcriptional profile. After MI, these differences became even more pronounced. Multiple cardiac cell populations, including fibroblasts and macrophages, exhibited an amplified inflammatory state, and T cells with a tissue-infiltrating effector phenotype were markedly more abundant in latently infected mice compared with naive controls. These immunologic changes were associated with signs of adverse cardiac remodeling during both the acute and chronic phases of MI, as evidenced by increased diastolic volumes in latently infected mice. Finally, we identified a correlation between virus-specific clonal T cell expansions and poorer post-MI outcome in patients. Taken together, by integrating experimental and clinical evidence, our study establishes the interplay among cytomegalovirus infection, myocardial immune cell dynamics, and post-MI repair.

Although laboratory mice kept in specific pathogen-free conditions are considered to closely mimic human cardiac function and tissue composition, their immune systems are not shaped by natural pathogen exposure, which can have a major impact on the study of immunologic processes.^40^ In experimental models, latent MCMV has been shown to change the leukocyte composition of the lungs, making them more resistant to bacterial^41^ and viral^42^ challenges. Furthermore, transcriptomic analyses of liver, spleen, and brain tissue of mice latently infected with a herpesvirus closely related to MCMV showed a clear proinflammatory change in gene expression patterns.^43,44^ However, the impact of cytomegalovirus exposure on the myocardial microenvironment remains poorly understood. Our findings reveal that latent MCMV infection produced distinct transcriptional and metabolic profiles in the heart, compared with noninfected animals. More specifically, during viral latency, we observed a downregulation of oxidative phosphorylation and respiratory pathways along with upregulated interferon responses, recapitulating mechanisms observed in mice overexpressing IFN-γ.^14^ In addition, a pronounced leukocyte infiltration began during the acute phase of infection and persisted above baseline levels even during MCMV latency. Among these infiltrating leukocytes, we particularly noted a population of CD8^+^ T cells that recognize an epitope derived from m139 MCMV protein and had all the phenotypic features of tissue-resident memory T cells (negative for intravascular CD45 staining and positive for CD69, CXCR6, CD38). It should be noted that even though MI could increase capillary permeability and thereby facilitate access of administered antibodies into the tissue, the short-term i.v. staining protocol utilized in our study minimizes nonspecific staining of nonvascular cells.^45–47^ This is supported by the observation that the vast majority of CD69^+^ CXCR6^+^ tissue-infiltrating CD8^+^ T cells did not show significant intravascular labeling (Figure 3). Tissue-resident T cells have been extensively characterized in various organs but seldom identified in cardiac tissue. A recent study by Kalinoski et al^48^ noted a population of cardiac myosin-specific Trm cells which were associated with an increased susceptibility of mice to develop immune checkpoint-induced myocarditis. Trm T cells were also found in the epicardial tissue of patients with atrial fibrillation.^49^ Nevertheless, to the best of our knowledge, our study is the first to define a population of virus-specific cardiac T cells with a Trm phenotype.

Considering the shifts in myocardial cell dynamics and the enhanced inflammatory milieu observed in MCMV-infected mice, we hypothesized that previous exposure to MCMV infection could impact post-MI scar formation and tissue remodeling. Thus, by combining MCMV infection and experimental MI models with scRNA-seq/scTCR-seq and cMRI phenotyping, our study reveals a virus-induced exacerbation of post-MI inflammation. This inflammatory response was observed across multiple myocardial cell subsets, including cardiac fibroblasts, myeloid, lymphoid, and endothelial cells, and was linked to adverse cardiac remodeling. Notably, a recent study published during the revision process reported on the impacts of influenza infection in myocardial injury models.^50^ The study shows that a type-I IFN signature linked to severe influenza infection fuels myocardial inflammation. In contrast to this recent publication, our study is tailored more to mild infection, though, mimicking natural exposure that does not require hospitalization. Our observations do not exclude that severe infection leading to hospitalization is a cardiac risk factor, especially during acute infection; however, dissecting these differences would be beyond the scope of the present article. In our study, CD8^+^ T cells poised with a Trm phenotype were particularly enriched in MCMV+MI mice. In line with our flow cytometry and RNA-sequencing findings, our scTCR-seq data further revealed a clonal expansion of MCMV-specific CD8^+^ T cells in the heart, suggesting that the enhanced inflammatory state observed in mice subjected to MCMV+MI may result from these virus-specific cells first infiltrating and then locally replicating in cardiac tissue. Previous studies have shown that persistent stimulation of effector T cells poised with a proinflammatory signature can fuel cardiac injury and adverse remodeling.^35,51–54^ Activated T cells secreting IFN-γ, IL-17, and TNF have been found to promote fibrosis development and adverse cardiac remodeling during the chronic phase of MI.^54^ Furthermore, Ilatovskaya et al^55^ reported that CD8^+^ T-cell-deficient mice have improved survival after MI despite having substantially elevated risk for cardiac rupture during its early stages. This increased cardiac rupture was associated with inadequate cross-linking of collagen fibers, a phenomenon which highlights the effect that T cells could have on cardiac fibroblasts.

Our flow cytometry and scRNA-seq analyses also showed pronounced alterations in macrophage composition and phenotype within the hearts of MCMV^+^ mice. Notably, viral infection was associated with a marked shift toward a CCR2^+^ macrophage population, and transcriptomic data showed an inflammatory signature among cardiac macrophages. CCR2^+^ macrophages are derived from hematopoietic progenitors and are replenished by circulating monocytes. On myocardial damage, these macrophages produce high levels of inflammatory cytokines and recruit neutrophils and monocytes, thereby inducing the generation of oxidative products in cardiac tissue.^56^ This macrophage population is associated with excessive inflammation and adverse cardiac remodeling,^57^ as blocking CCR2 or selectively depleting CCR2^+^ macrophages resulted in improved cardiac function and smaller LV sizes after experimental MI.^58^ It is, therefore, plausible that MCMV infection primes the cardiac macrophage population toward a heightened proinflammatory state, which, following MI, may exacerbate inflammation and ultimately contribute to adverse cardiac remodeling. Cardiac fibroblasts are extensively regulated by inflammatory cues received from their environment^59^ and an inflammatory milieu generated by latent cytomegalovirus infection could lead to pathological collagen deposition, ultimately resulting in adverse remodeling. Consistent with this notion, we have also observed an increased gene expression signature associated with interferon and TNF pathways in cardiac myofibroblasts of MCMV+MI mice. It is important to note that our scRNA-seq analysis of the less abundant cell populations focused on cell type phenotypic characterization, rather than inferring differential gene expression signatures between cytomegalovirus-infected and noninfected animals, which would require statistical interpretation and power. This includes CD8^+^ T cells from noninfected infarcted mice that were very rare relative to the diverse subsets of immune cells included in the pooled CD45^+^ scRNA-seq library from myocardial scars. Nevertheless, the obtained signatures and phenotypes match our FACS analyses and are also consistent with other scRNA-seq data sets of total CD45^+^ cells purified from myocardial scars.^60,61^

Cytomegalovirus has been identified in cardiac tissue through both postmortem human studies^22^ and experimental mouse models.^25^ Consistent with these observations, we noted active viral replication within cardiac tissue for up to 9 days following infection (Figure 1C and 1D). This occurred in the absence of obvious cardiac necrosis, tissue damage, or long-term compromised myocardial functions. These observations are in agreement with a recent study by Cirnu et al^57^ who, in the context of congenital cardiomyopathies, found that MCMV infection did not affect baseline cardiac function in wildtype mice, as determined by cMRI. On the contrary, we showed that MCMV infection induced long-term alterations of the cardiac-resident immune cell compartment. Several epidemiological and other studies have linked latent cytomegalovirus infection to poorer outcomes after cardiovascular events.^18,21,62^ Supporting this association, our analysis of a cohort of patients with MI revealed that elevated anti-cytomegalovirus antibody titers correlated with diminished cardiac function 12 months after the initial event. Moreover, TCR sequencing uncovered a notable overlap between cytomegalovirus-annotated TCRs and those expanded in patients who showed inadequate improvement in LVEF post-MI, an overlap not found with EBV-annotated TCRs. In line with these data, the retrospective analysis of the FRACTAL placebo cohort further confirmed that cytomegalovirus-seropositive STEMI patients exhibited larger infarcts and sustained expansion of circulating CD8^+^ and CD4^+^ CX3CR1^+^CD28^−^ cells after MI. These dynamics further support the connection between cytomegalovirus infection and adverse cardiac outcomes. Our animal studies recapitulated these clinical observations, as echocardiography and cMRI assessments, performed 5 and 56 days after MI, respectively, determined that MCMV^+^ mice had worse adverse cardiac remodeling compared with infarcted controls maintained under specific pathogen-free conditions. This was associated with increased IFN-γ-dependent inflammatory signatures and a strong increase in effector CD8^+^T cells in the heart during MI.

Importantly, the cardiac T cells that we identified were clearly distinct but coincided with the presence of CX3CR1^+^ inflammatory memory T cells that are characteristically expanded in the peripheral blood of a large fraction of patients with latent cytomegalovirus infection.^63^ Intriguingly, these cells correlated with infarct size in the FRACTAL study, suggesting that their measurement in PBMCs warrants evaluation as a potential marker to stratify MI patients. Although these cells may serve as a marker that correlates with an altered systemic and cardiac-resident immune compartment, more work is required to mechanistically dissect the potential contributions of circulating versus cardiac T cells identified here, or the altered myeloid compartment. Intriguingly, Trm cells are emerging as critical sentinels of tissue damage and infection and are proposed to rapidly amplify and orchestrate local inflammation. Development of novel genetic tools will be required to dissect the complex cellular interactions of immune cells in a selected tissue, such as the heart. In addition, our preliminary data suggest a potential viral reactivation on CD8^+^ T cell depletion in MCMV+MI animals, together with previous findings^64^ highlight the limitations and complexity of currently available approaches. Therefore, future studies may also focus on potential interventions, for example, by blocking inflammatory mediators that could support rapid clinical translation. Prioritizing such potential therapeutic targets will benefit from clinical investigations of CMV^+^ versus CMV^−^ STEMI patients, which we expect to be stimulated by the report of our findings. It is important to emphasize that our clinical data revealed an association between cytomegalovirus-specific TCR motifs and disease outcome, whereas the cytomegalovirus serostatus did not differ between good and poor healers. These findings suggest that the critical factor determining patients’ outcomes is the extent of cytomegalovirus-driven T-cell expansions, rather than cytomegalovirus infection per se. These observations provide a rationale for more refined diagnostic strategies to stratify patients based on virus-specific TCR signatures, rather than cytomegalovirus serostatus alone.

To conclude, here we present an in-depth molecular and cellular analysis of changes induced in cardiac tissue by a common herpesvirus infection. We experimentally explored and validated long-standing epidemiological data linking cytomegalovirus infection with worse cardiac outcomes after cardiovascular events. Our clinical data confirmed our murine findings, revealing a potential mechanistic link between cytomegalovirus-induced immune changes and adverse cardiac remodeling in patients with MI. These results position cytomegalovirus serostatus as a clinically relevant modifier of post-MI healing, with potential utility in risk stratification and personalized management. Future studies should focus on prospective validation of cytomegalovirus and associated T-cell expansions as biomarkers to refine prognostication and guide therapeutic decision-making in ischemic heart disease.

## Article Information

### Acknowledgments

The authors thank Lisa Popiolkowski, Dijana Rumora, Cristina Paulović, Antonija Šarlija, Andrea Leupold, and Elena Vogel for their skillful technical assistance. The authors also thank the Core Unit SysMed at the University of Würzburg for excellent technical support, RNA sequencing data generation, and analysis. The authors also thank the National Institutes of Health Tetramer Core Facility for providing m139, p79, and influenza virus nucleoprotein (NP) tetramers. The authors would like to thank Heiko Adler and Simone Backes for providing MHV-68 (murine gammaherpesvirus 68) and Influenza WSN stocks, respectively. The figures were prepared with the help of Servier Medical Art (SMART) and Biorender (Grapical abstract; Created in BioRender. Brizić, I. (2026) https://BioRender.com/q82m91z).

### Author Contributions

This study was conceptualized by M. Šustić, I. Brizić, G. Gasteiger, and G. Campos Ramos. The methodology was developed by E. Dapergola, M. Šustić, M. Srivastava, P. Arampatzi, D. Ashour, M. Cokarić Brdovčak, J. Materljan Franki, B. Lisnić, E. Bellulovich, K. Kashofer, M. Terekhov, K. Le Gouge, E. Mariotti-Ferrandiz, P.P. Rainer, T. Krammer, A.-E. Saliba, S. Jonjić, U. Hofmann, S. Frantz, I. Brizić, G. Gasteiger, and G. Campos Ramos. The investigation was conducted by E. Dapergola, M. Šustić, D. Ashour, M. Cokarić Brdovčak, J. Materljan Franki, B. Lisnić, E. Bellulovich, V. Stangl, L. Rech, M. Terekhov, T. Krammer, K. Le Gouge, S. Plein, Y. Ekinci, C. Cochain, and I. Spyridopoulos. The funding was provided by M. Šustić, P.P. Rainer, I. Brizić, G. Gasteiger, G. Campos Ramos, I. Spyridopoulos, and S. Jonjić. The project was administered by S. Jonjić, I. Brizić, G. Gasteiger, and G. Campos Ramos. The study was supervised by S. Jonjić, I. Brizić, G. Gasteiger, and G. Campos Ramos. The original draft was written by E. Dapergola, M. Šustić, D. Ashour, and G. Campos Ramos. The article was reviewed and edited by all authors.

### Disclosures

None.

### Supplemental Material

Supplemental Methods

Figures S1–S9

Tables S1–S3

References 65–76

Major Resources Table

## Supplementary Material
