# Supplementary material for: Cytomegalovirus Latency Fuels Inflammation Following Myocardial Infarction
Source: Circ Res. 2026 Sep 14;139(9):e327799. doi: 10.1161/CIRCRESAHA.125.327799 (PMC13649278; doi:10.1161/CIRCRESAHA.125.327799)
Supplement: Supplementary file 1 [file res-139-e327799-s001.pdf]

Supplemental Material

Cytomegalovirus latency fuels inflammation following myocardial infarction

Eleni Dapergola[1,#], Marko Šustić[2,#], DiyaaElDin Ashour[3,4,#], Ebram Tharwat Melika[3,4,18], Maja Cokarić Brdovčak[5], Jelena Materljan Franki[5], Mijo Golemac[6] , Berislav Lisnić[5], Ema Bellulovich[5], Valérie Boivin-Jahns[7], Tobias Krammer[8], Kenz Le Gouge[9], Lavinia Rech[9], Verena Stangl[10], Karl Kashofer[11] Peter P. Rainer[9,14], Roland Jahns[15], Encarnita Mariotti-Ferrandiz[12,13], Hamid Marwan[4], Maxim Terekhov[4], Mohammadreza Keshtkar[4], Antoine-Emmanuel Saliba[8], Clement Cochain[16,17], Panagiota Arampatzi[19], Mugdha Srivastava[19], Sven Plein[20], Yasemin Ekinci[21], Ioakim Spyridopoulos[21,22], Stipan Jonjić[5], Ulrich Hofmann[3,4], Stefan Frantz[3,4], Ilija Brizić[5,§], Georg Gasteiger[1,§], and Gustavo Campos Ramos[3,4, §]

[1] Würzburg Institute of Systems Immunology, Max Planck Research Group at the Julius-Maximilians-University Würzburg, Würzburg, Germany

[2] Department of Internal Medicine, Faculty of Medicine, University of Rijeka, Rijeka, Croatia

[3] Department of Internal Medicine I, University Hospital Würzburg, Würzburg, Germany

[4] Comprehensive Heart Failure Center, University Hospital Würzburg, Würzburg, Germany

[5] Center for Proteomics, Faculty of Medicine, University of Rijeka, Rijeka, Croatia

[6] Department of Histology, Faculty of Medicine, University of Rijeka, Rijeka, Croatia

[7] Institute of Pharmacology and Toxicology, University of Würzburg, Würzburg, Germany

[8] Helmholtz Institute for RNA-based Infection Research, Helmholtz Centre for Infection Research, Würzburg, Germany

[9] Division of Cardiology, Department of Internal Medicine, University Heart Center, Medical University of Graz, Graz, Austria BioTechMed Graz, Graz, Austria

[10]Department of Internal Medicine, Landeskrankenhaus Südweststeiermark, Wagna, Austria (V.S), Diagnostic and Research Institute of Pathology, Medical University of Graz, Austria (V.S)

[11]Diagnostic and Research Institute of Pathology, Diagnostic and Research Center of Molecular BioMedicine, Medical University of Graz

[12] Immunoregulation-Immunopathology-Immunotherapy, UMRS959, Sorbonne Université, INSERM, Paris, France

[13]Institut Universitaire de France, Paris, France

[14] Department of Medicine, St. Johann in Tirol General Hospital, St. Johann in Tirol, Austria

[15]Interdisciplinary Bank of Biomaterials and Data Würzburg (ibdw), University Hospital Würzburg, Würzburg, Germany

[16]Université Paris Cité, Inserm, PARCC, 56 rue Leblanc, 75015 Paris, France

[17]Institute of Experimental Biomedicine, University Hospital Würzburg, Würzburg, Germany

[18]Department of Biochemistry, Faculty of Pharmacy, Minia University, Minia, Egypt

[19]Core Unit Systems Medicine, University of Würzburg, Würzburg, Germany

[20]Leeds Institute of Cardiovascular and Metabolic Medicine, University of Leeds, United Kingdom of Great Britain & Northern Ireland

[21]Newcastle University, Translational Research Institute, Vascular Biology and Medicine Theme, Faculty of Medical Sciences

[22]Freeman Hospital, Cardiology, Newcastle-Upon-Tyne, United Kingdom of Great Britain & Northern Ireland

[#] These authors contributed equally.

[§] **Corresponding authors**:

Prof. Dr. Ilija Brizić

Center for Proteomics, Faculty of Medicine

Brace Branchetta 20

51000, Rijeka, Croatia

Email: ilija.brizic@uniri.hr

Prof. Dr. Georg Gasteiger

Institute of Systems Immunology

Building E6

Versbacher Str. 9

D-97078 Würzburg, Germany

Email: georg.gasteiger@uni-wuerzburg.de

Prof. Dr. Gustavo Ramos

Immunocardiology Lab

University Hospital Würzburg

Am Schwarzenberg 15, Haus A15

D-97078 Würzburg, Germany

Phone: +49 931 201 46477

Fax: +49 931 201 46485

Email: Ramos_G@ukw.de

Supplemental material Content

Supplemental methods

Supplemental Figures S1-S9

Supplemental table S1-3

Methods

Experimental myocardial infarction

Myocardial infarction was induced by placing a permanent suture ligature around the left anterior descending coronary artery (LAD) to achieve permanent coronary occlusion, according to published guidelines[65]. Mice were anesthetized with isoflurane, intubated for mechanical ventilation and subjected to thoracotomy under aseptic conditions. For this purpose, the mouse's skin was first shaved and sterilized, followed by a surgical incision to expose the third intercostal space. A Ringer lactate-soaked piece of sponge was used for lung displacement and the LAD of the mice was permanently ligated with a 6/0 perma-hand silk thread. This specific step was excluded in sham operated mice. After the ligation was finished the chest cage and skin were sutured using a 5/0 and 6/0 Prolene thread respectively, isoflurane concentration was reduced to 0.5% and mice were extubated. Body temperature was controlled during the operation, and the mice received pain management with buprenorphine s.c. 0.1 mg/Kg body weight (Buprenovet, Bayer Vital, Leverkusen, Germany) starting one hour before and for at least 3 days (twice daily) after operation. The surgeries were performed by an experimenter who was blinded to the different treatments.

Mice

For experiments conducted in Würzburg, male and female C57BL/6J mice were purchased from Charles River Laboratories and were kept in individually ventilated cages under pathogen-free conditions in animal facilities. For the sequential infections mice were used at the age of four weeks, while for the individual infections and the procedures with MCMV and MI, mice were used after the age of eight weeks. The CD8 depletion experiments were performed using male $CD8^{iDTR}$ mice, generated by crossing C57BL/6-*Gt (ROSA)26Sor*$^{tm1(HBEGF)Awai}$/J (Jackson strain #0079000) with C57BL/6-Tg (Cd8a-cre)1Itan/J (Jackson strain #008766). All animals were kept under a 12h-12h light-dark cycle at 22°C and relative humidity (40–50%) with ad libitum access to normal food pellets and sterile drinking water. For experiments conducted in Rijeka, adult (6-12 weeks old) female wild-type C57BL/6J mice (strain #000664) were used in experiments. All mice were housed and bred under the conditions mentioned above at the animal facility of the Faculty of Medicine, University of Rijeka, The Animal Welfare Committee at the University of Rijeka, Faculty of Medicine, and The National Ethics Committee for the Protection of Animals Used for Scientific Purposes (Ministry of Agriculture) approved all animal experiments. Randomisation was performed prior to infection by sequentially assigning each mouse to one of two cages at random until no animals remained.

Viruses and viral titratrion

Experiments in Rijeka were conducted using tissue cell culture derived Mck2-repaired Δm157MCMV strain. The backbone of this virus is the Mck2-repaired BAC-encoded MCMV strain (Smith strain), clone pSM3fr-MCK-2-fl 3.3[66], and genetic modifications was done using en passant mutagenesis[67].

Virus stocks and heart homogenates were titrated on mouse embryonic fibroblasts (MEFs) using standard plaque assay procedure, described in detail previously[68]. Before taking the hearts for plaque forming assay procedure, the mice were perfused with sterile PBS. Adult mice were immunized intravenously with $2x10^5$ plaque forming units (PFU).

Similarly, experiments performed in Würzburg utilized a wildtype Smith strain of MCMV, directly propagated from cell culture. C57BL/6 mice were infected intraperitoneally (i.p.) with 1 x 10^5 PFU before being subjected to experimental MI. For the sequential infections mice were infected with 10^5 PFU of MHV-68 intranasally (i.n.) at four weeks of age, and then at 2-week intervals with 10^5 PFU of MCMV i.p. and 10^3 PFU of influenza strain WSN i.n. For the individual infections three groups of eight-week-old male C57BL/6 mice were subjected to either MHV-68 i.n., WSN i.n. or MCMV i.p. and were sacrificed four weeks later.

CD8 T cell depletion

$CD8^{iDTR}$ mice were infected at four weeks of age and four weeks later subjected to MI. Diphtheria toxin (Dtx; 1 µg) was administered intraperitoneally on days −2 and −1 before MI, and on days 2 and 4 after MI.

Echocardiography

Echocardiographic assessments were conducted by a blinded experimenter on a Vevo 1100 instrument with a 30-MHz probe, specifically developed for mice studies. Mice were kept under slight anesthesia with isoflurane (0.5-1.5% vol/vol O_2) on a heated bed (39°C) and images were taken on the short axis at the apex, midpapillary (B and M mode) and basal levels (only B mode) and on the long axis (B and M mode) to define the infarct border zones. Analyses were performed using the Vevo LAB 3.2.6 software and the infarct size was measured in the longitudinal axis (B mode) by calculating the quotient from the heart size and the akinetic myocardium. Only mice with an infarct size strictly above 30% were included in the analysis as recommended by guidelines for in vivo models of MI[69].

Cardiac Magnetic Resonance Imaging

Mice were initially anesthetized with approximately 4% isoflurane (v/v) in an induction chamber until loss of consciousness. Anesthesia was maintained with 2.0–2.5% isoflurane delivered via a nose cone, using a carrier gas (oxygen) at a flow rate of approximately 1 L/min. Monitoring and cardiac gating were done using SAII gating and monitoring system Model 1025 (Stony Brook, NY, USA). ECG signals were detected using 3-lead golden-plated electrodes fixed at the paws, temperature was controlled using a rectal sensor, and breathing signals were detected using a breathing pad sensor. Warming of a mouse during the scan procedure was performed using a water-heated warming pad[70].

All measurements were performed using a preclinical 7T MRI scanner PharmaScan (Bruker BioSpin, Ettlingen, Germany). The bird cage coil with 40 mm inner diameter was used. CINE MRI images were acquired using both ECG- and breathing-gated gradient echo sequence and self-gated variant (“IntraGate” ™). The sequence parameters were TR/TE = 5/1.4ms, flip-angle = 20^0, in-plane pixel resolution = 0.18 mm^2, slice thickness = 1mm. The acquisition time was 90-120 seconds per slice (depending on breathing and heart rate) using 3 signal averages. The stack of 6 to 8 short-axis view slices was acquired using 20 cardiac phases. Additionally, the single slice of 4-chamber (long-axis) view was measured in order to visualize remodeling of the left heart with an increased number of averages NA=4 and a spatial resolution, providing 0.16x0.16x0.8mm voxel size.

The evaluation of cardiac function parameters was done using. “MedisSuite MR“software („Medis Medical Imaging “, Leiden Netherlands). Manual segmentation of the left ventricle (without using automated segmentation tools) has been performed. The evaluated cardiac function parameters were: ejection fraction, end-systolic and end-diastolic volumes, stroke volume, cardiac output, and end-systolic and end-diastolic myocardial mass (left ventricle).

Flow Cytometry and cell sorting

For experiments conducted in Würzburg, mice were injected intravenously via the tail vein with 2µg anti-CD45 in 150µl PBS 4 minutes before euthanasia and organ collection[44-46]. Biotin-conjugated anti-CD45 (clone 104) was used for sequencing run 1 and AF700-conjugated anti-CD45 (clone 13/2.3) for runs 2–3. All other experiments used BV650-conjugated anti-CD45 (clone 30-F11). Hearts were perfused with PBS + Heparin (10%) and the infarcted tissue including the border zone was carefully isolated, weighted, chopped in pieces and eventually digested in Collagenase II (1,000 IU/ml Worthington, Cat #LS004176) + DNAse I for 30 minutes in a 37°C prewarmed water bath. Next, the digestion mix containing the tissue was passed through a 70µm cell strainer, washed with HBSS/BSA medium and transferred to a 96 U-well-bottom plate for staining. Cells were stained at 4°C for 15 minutes in the dark with either Aqua Zombie Fixable viability dye (Biolegend Cat# 423102) for identification of myeloid cells or with Zombie NIR™ Fixable viability dye (BioLegend, Cat# 423106) for T cell analysis including anti-mouse CD16/CD32 antibody (clone 93, BioLegend, Cat# 101302) to block Fc receptors and minimize non-specific binding. Following viability staining class I MHC (H2-kb) MCMV-tetramer M139$_{419–426}$ (TVYGFCLL) purchased by the NIH tetramer core facility was added to the cell suspension for the detection and phenotyping of virus-specific CD8^{+} T cells. Cells were incubated for 20 minutes at 4°C and then stained with primary antibodies for another 20 minutes at 4°C. Finally, cells were resuspended in HBSS/BSA and acquired on a five-laser Cytek Aurora full-spectrum flow cytometer. Representative FACS plot images were selected for the main and the supplemental figures after analysis, from samples with measurements closest to the respective group mean. Gating strategy was designed based on biological negative-control populations and where applicable unstained samples.
Similar protocol was used in experiments conducted in Rijeka. Briefly, mice were sacrificed using cervical dislocation, perfused with cold PBS-heparin solution and the hearts were obtained. Cardiac tissue was mechanically minced and afterwards enzymatically digested with Liberase TM (25µg/mL) for 35 minutes at 37°C. After digestion the tissue was filtered through 70µm filter mesh. Samples were than washed with PBS and subsequently stained with Fixable Viability Dye eFluor™ 780 (Thermo Fischer Scientific) for 15 minutes. After washing step with FACS buffer, the samples were surface-stained with conjugated antibodies in the presence of Fc blocking antibody (anti- CD16/CD32, clone 2.4G2, BD Pharmingen). The conjugated antibodies used (Thermo Fisher Scientific unless indicated): anti-CD45.2 (clone 104), anti-CD3 (clone 145-2C11), anti CD8 (clone 53-6.7), anti-CD4 (clone RM4-5), anti-CD62L (clone MEL-14), anti-CD44 (clone IM7), anti-CD11b (clone M1/70), anti-CD64 (clone X54-5/7.1), anti-MHC II (clone M5/114.15.2), anti-Ly6C (clone HK1.4), anti-CCR2 (R&D systems, clone# 475301).
Sorting of cardiac CD45^{+} and CD45^{-} cell populations was performed using a modified protocol from Skelly et al[71]. Heart tissue was digested, and a single cell suspension was prepared as described above. Cells were stained with Fixable Viability Dye eFluor™ 780 (Thermo Fischer Scientific) for 15 minutes, washed and stained with the tetramer M139$_{419–426}$ (APC for run 1 or PE for seq. runs 2-3), anti-CD45 (clone 30-F11) and anti-CD31 (clone 390) antibodies in sterile azide-free MACS buffer (0.5% BSA, 2mM EDTA in PBS). Mouse TotalSeq™-C antibodies were added to the antibody mix as described in Supplemental Table 1. Cells were then stained in Calcein violet dye (4 µg/ml, ThermoFisher Scientific) for 10 minutes at 37°C and resuspended in sterile azide-free MACS buffer before sorting using a FACS Aria II cell sorter (BD biosciences). Three experimental sessions were performed for sorting and scRNA/TCR sequencing. Three populations of cells were sorted from each mouse: 1) CD45^{+} CD31^{-} immune cells, 2) CD31+ CD45^{-} endothelial cells and 3) CD31^{-} CD45^{-} resident mesenchymal cells (Supp. fig. S2A). Two sequencing libraries were prepared from each session; one library from the pooled CD45+ immune cells, and one library of non-immune cells (mixed 1:1 from CD31^{+} ECs, and CD31^{-} non-ECs sorted populations).

RNA isolation and sequencing

For RNA isolation performed in Rijeka, mice were sacrificed using cervical dislocation, perfused with cold PBS-heparin solution and approximately the same size of mid-ventricle tissue was taken in all the samples. RNA was isolated with NucleoSpin® TriPrep (Macherey-Nagel®) isolation kit according to manufactures instructions. Agilent Bioanalyzer 5300 and Agilent High Sensitivity RNA Analysis Kit (DNF-472) were used to estimate sample quality and determine the quantity of isolated RNA. RNA-seq libraries were generated using the TruePrep DNA Library Prep Kit for MGI (VAZYME) as per the manufacturer's recommendations. Single-stranded circle DNA molecules where then replicated via rolling cycle amplification, and a DNA nanoball (DNB) which contain multiple copies of DNA was generated. Sufficient quality DNBs are then loaded into patterned nanoarrays using high-intensity DNA nanochip technique and sequenced through combinatorial Probe-Anchor Synthesis (cPAS) on DNBSEQ (MGI) sequencing platform in a single end 1x75 base pair run.

For the RNA extraction performed in Würzburg, the heart was homogenized in PBS (#L 182-50) using Qiagen tissue homogenizer and disposable probes (Qiagen #990890) in RLT supplemented with β-mercaptoethanol lysis buffer using Qiagen all prep kit (#80004) according to manufacturers' instructions. RNA concentration and purity were routinely checked using NanoDrop 2000 (Thermo Scientific™ ND2000).

Bulk RNA sequencing analysis

In Rijeka,RNA-Seq data analysis was performed essentially as previously described[69], with minor modifications specific to the particular experimental system employed in this study, and using a workstation equipped with an AMD Ryzen 9 9950X3D, NVIDIA GeForce RTX 4080 SUPER, 96 GB of DDR5 RAM, and running R v4.5.1 and RStudio Desktop (2025.09.0+387) [3] under Ubuntu 24.04.02 LTS (www.ubuntu.com). Operating system, drivers, and all other software packages and tools were updated to the latest versions during analysis. Software packages were used according to their respective manuals, vignettes, or materials published by the Harvard Chan Bioinformatics Core[72].

Initial quality control of the raw sequencing reads obtained from the sequencing core facility was performed using FastQC[73], and the sequence composition of the FASTQ files was then evaluated with FastQScreen v0.16.0 to rule out the presence of technical artefacts and contaminating sequences in the sequencing libraries. In addition, before read mapping and differential gene expression analysis, raw sequencing reads were aligned to the mouse genome sequence[74] using STAR[75] followed by the post-alignment quality control round using QoRTs package.

Following quality control, gene-level expression values for all samples, estimated and imported into R using salmon and tximport, were used to construct the DESeqDataSet object with DESeq2. Principal component analysis was performed with PCAtools and differentially expressed genes were identified using the Wald test from the DESeq2 package. Genes with Benjamini-Hochberge adjusted p values smaller than 0.01 were considered to be significantly differentially expressed between experimental conditions/groups. Gene ontology over-representation analysis (GO-ORA) of the list of differentially expressed genes was performed using clusterProfiler and GOplot packages. Heatmaps of gene expression were generated using complexHeatmap package whereas the volcano plot, and the results of the PCA and GO-ORA were visualized using packages including tidyverse, RColorBrewer, colorspace, extrafont and ggtext.

In Würzburg, RNA quality was assessed using a 2100 Bioanalyzer with the RNA 6000 Nano kit (Agilent Technologies), ensuring all samples had an RNA integrity number (RIN) ≥7.5. DNA libraries suitable for sequencing were prepared from 200 ng of total RNA using oligo-dT capture beads for poly-A enrichment, following the TruSeq Stranded mRNA Library Preparation Kit (Illumina) protocol at half volume. After 15 cycles of PCR amplification, the size distribution of the barcoded DNA libraries was estimated to be ~310-320 bp via electrophoresis on Agilent DNA 1000 Bioanalyzer microfluidic chips. Sequencing of pooled libraries, spiked with 1% PhiX control library, was conducted at 25 million reads per sample in single-end mode with a 100.

Single cell RNAseq library construction

Infarcted hearts from naïve and MCMV-infected C57BL/6J mice were collected five days after MI and dissociated into single-cell suspensions using a modified protocol based on Skelly et al.[71] Both hematopoietic (viable Calcein$^+$ CD45$^+$ cells) and non-hematopoietic populations (viable Calcein$^+$ CD45$^-$ CD31$^+$ and CD45$^-$ CD31$^-$ cells) were isolated to enable transcriptional profiling of immune and non-immune cardiac cell populations. Sample multiplexing was performed using oligo-tagged hashtag antibodies, and three independent single-cell sequencing experiments were conducted.

Single cell RNA-sequencing Chromium controller was used to partition single cells into nanolitre-scale Gel Bead-InEmulsion (GEMs) and Chromium Next GEM Single Cell 5' v1.1 kits for reverse transcription, cDNA amplification and library construction (10x Genomics). A SimpliAmp Thermal cycler was used for amplification and incubation steps (Applied Biosystems). Libraries were quantified by a Qubit 3.0 fluorometer (ThermoFisher) and quality was checked using a 2100 Bioanalyser with high sensitivity DNA kit (Agilent) in paired-end mode to reach at least 60000 reads per single cell for gene expression and 6000 reads for the T cell receptor repertoire, hashtags and CITE-seqs. The Cell Ranger Software suite (version 7.0.1, 10x Genomics) was used for sequence alignment, barcode processing and sample demultiplexing.

Single cell RNAseq and data preprocessing

Gene count matrices derived from Cell Ranger was analysed using Seurat package (version 5.0.1). For each sample, gene expression, hashtag oligonucleotide (HTO), and CITE-seq antibody signals were loaded separately. Sample de-multiplexing was performed using Seurat's HTODemux (positive quantile of 0.99), assigning singlet, doublet, or negative status to each cell. After removing doublets and negative cells, Singlet cells were filtered to exclude those with fewer than 200 detected genes, >5% mitochondrial RNA content, or total UMI counts > 35,000 for CD45$^+$ datasets and > 25,000 CD45$^-$ datasets. RNA assays from each sample were log-normalized using Seurat's default approach, while CITE-seq and HTO assays underwent centered log-ratio (CLR) normalization. Next, individual libraries were merged into a joint Seurat object, with library origin annotated in the metadata for downstream QC and batch assessment (Fig. S2A). To improve clustering accuracy and minimize technical artifacts, dataset was cleaned by regressing out the effects of: a) genes induced by warm enzymatic digestion, as annotated previously[76] and b) ribosomal genes during the ScaleData step in Seurat. These genes were specified in the vars.to.regress argument during data scaling, which does not remove these genes from the expression matrix but rather equalizes their expression in scaled data which is used for downstream clustering and dimensionality reduction. Principal component analysis was performed on the variable genes (n=70 PCs) and clustering was conducted using FindNeighbors and FindClusters (resolution 0.4), followed by UMAP visualization on the first 70 PCs. Marker genes for each cluster were defined using the FindAllMarkers function (log2 fold-change threshold 1, only positive markers). Marker lists for each cluster were cross-referenced against published single-cell cardiac datasets, including

Forte et al.[30], for annotation and validation. No batch integration was performed. All libraries were prepared by the same operator, on the same instrument, and using the same protocol to minimize technical variance. The absence of batch effects was verified through dimensionality reduction (Fig. S2C), which revealed extensive overlap of annotated clusters across experiments, with no discrepancies between different batches.

Gene set enrichment analysis of selected cell clusters

Within each lineage-defined subset, differential gene expression analysis between MCMV+MI and MI conditions was performed using the FindMarkers function in Seurat. Upregulated genes with adjusted p-values <0.05 were used to generate a gene list ranked by effect size. Hallmark gene sets from the Molecular Signatures Database (MSigDB, category "H") for mouse were retrieved via the msigdbr R package (version 10.0.2). Gene symbols were matched to the ranked list of cluster-specific differentially expressed genes. The GSEA function from the clusterProfiler R package (version 4.6.2) was used for gene set enrichment. Multiple testing correction was applied using the Benjamini-Hochberg method, and significantly enriched hallmark pathways (FDR<0.05) were visualized as dot plots.

Core genes upregulated in the GSEA enriched pathways were plotted as heatmaps using the ComplexHeatmap R package. Only genes that are part of the core enrichment gene set (as designated by clusterProfiler), with log2 fold-change > 0.4 and expressed in the upregulated subset > 20% were selected. Up to four representative core genes were selected per cell type based on effect size. Genes were grouped according to their pathway annotation, and the averaged expression values were compared between MCMV+MI against MI.

Single cell TCR sequencing data analysis

T cell receptor (TCR) sequencing data from the three independent 10x Genomics libraries were processed using the scRepertoire R package (version 2.0.8). Filtered contig annotation files were imported for each library, and the paired alpha and beta chain information were integrated based on matching 10x barcodes using the combineTCR function and incorporated into the Seurat object using combineExpression. Clonotypes were defined based on CDR3 amino acid sequences, and clonal expansion categories were defined based on the frequency of each clonotype within the dataset. Next, the beta chain CDR3 (CDR3β) sequences were extracted from TCRs observed in at least 3 cells, representing expanded clonotypes. To investigate the potential antigen specificity of the expanded TCRs, publicly available MCMV-annotated TCR β-chain sequences from the McPAS-TCR database, which compiles experimentally validated TCRs against common pathogens were integrated with the expanded CDR3β sequences from the dataset. A curated list of TCRs derived from an immune checkpoint inhibitor (ICI) myocarditis study[34] was also used to probe for cardiac antigen specific TCRs. The combined dataset was analyzed using the turboGliph package (version 0.99.2), which is an R implementation of the GLIPH2 (Grouping of Lymphocyte Interactions by Paratope Hotspots 2) algorithm[32,33]. GLIPH2 identifies conserved CDR3β motifs within a given dataset and clusters TCRs into hubs of matched CDR3β motifs. CDR3βs of a minimum length of 8 amino acids and a motif boundary size of 3 was used, Fisher's exact test was used to identify motifs in the core region of the CDR3 locus of the beta chain that are at least 3 aa in length and are significantly enriched (P-value < 0.01) against a reference repertoire of 78,116 non-disease-associated mouse TCRs. The resulting GLIPH2 clustering output was used to construct TCR similarity networks where nodes represent individual CDR3β sequences and edges denote shared sequence motifs. Only clusters containing ≥5 sequences were visualized in the network.

Patient cohorts

To interrogate a possible association between CMV infection and MI outcomes in patients, we retrospectively stratified and analyzed data from two complementary cohorts where peripheral blood (Würzburg) and myocardial tissue (Graz) samples were available. For the blood samples from Würzburg, the ETiCS study was approved by the ethics commission of the University of Würzburg (186/09) and met the criteria of the Declaration of Helsinki. All patients provided written informed consent. Additionally, the use of human tissues collected in Graz, is conformed with legal and institutional requirements and was approved by the ethics committee of the Medical University of Graz (31-288 ex 18/19).

Würzburg cohort

Peripheral blood samples available from the ETiCS cohort (Etiology, Titer-Course and effect on Survival of cardiac autoantibodies-study, Würzburg arm) were retrospectively analysed. The study enrolled 150 patients including individuals of both sexes. The stratification of patients was conducted with following inclusion criteria: ST-segment elevation in at least two contiguous leads (≥0.1 mV in limb leads, ≥0.2 mV in chest leads), or newly documented left bundle branch block accompanied by typical symptoms of myocardial infarction, no prior history of myocardial infarction, confirmation of acute myocardial infarction by coronary angiography and new onset chest pain within the previous 7 days. Patients were excluded from the study if they fulfilled following criteria: malignancy or any other disease limiting the expected survival to less than 1 year, end-stage renal failure or chronic haemodialysis, significant rheumatic or autoimmune disease requiring immunomodulatory therapy, congenital neuromuscular disorders and myasthenia gravis, grave's disease, ongoing drug or alcohol abuse and pregnancy. From 150 patients, 54 had LVEF <50% at baseline. 38 samples from 54 patients with complete cMRI follow-up at 12 months were grouped into "good healer" or "poor healer" categories, based on whether patients showed a ΔLVEF improvement >13% at 12-month follow-up compared to baseline. RNA of cryopreserved whole blood samples collected at hospitalization were selected for bulk TCR RNA sequencing. 28 samples passed the quality control to be sequenced (19 poor healers, and 9 good healers), as described in Le Gouge et al.,[36]. The sequenced samples were from predominantly male patients (≈75–80%) with a mean age of approximately 60 years.

Graz cohort

Human myocardial tissue was derived from patients that underwent routine autopsy at the Medical University of Graz between 2008 and 2018. In total, 864 autopsies were found with the diagnosis "myocardial infarction" in the pathology report. Of these, patients were selected that had been treated at the Division of Cardiology for acute coronary syndromes (302 patients). Patients were excluded if they had active cancer, acute infections, known autoimmune disease, cardiac amyloid, fever, immunosuppression or missing reports. Cases were included if FFPE tissue blocks available in our tissue bank (77 patients) and underwent histologic examination (H&E and IHC for CD3). Patients were included if tissue sections demonstrated at least moderate infiltration with $CD3^+$ T cells. Finally, tissues from 23 patients (13 female, 10 male) were selected. All had clinical and/or macroscopic and histologic evidence of MI on autopsy. Median time of MI to death was 6.5 days (range 0-52 days) and mean age was 68 years (range 39-87 years). Ten of these patients had suffered from prior MI.

FRACTAL clinical trial

We conducted a retrospective analysis of the placebo cohort from the FRACTAL trial (EudraCT 2021-001354-53), which was a phase IIa, randomised, double-blind, multi-centre study designed to evaluate the safety and cardio-protective effects of CX3CR1 inhibition in STEMI patients undergoing primary percutaneous coronary intervention (PPCI). The placebo arm

(n=30) consisted of participants aged 18-75 who received an investigational product identical in appearance to the active drug, beginning with a 6-hour intravenous infusion (Kleptose HP) followed by a three-day oral regimen administered every eight hours. Participant allocation followed a minimisation scheme to balance groups based on gender, TIMI flow pre-reperfusion (0, 1, or 2), and time from pain onset. Specific eligibility criteria, especially the thresholds used to identify "high responder" participants based on lymphocyte subsets, were derived from parameters previously published by Loh et al[24]. This retrospective analysis utilised the study's comprehensive 90-day follow-up data, which included longitudinal assessments of cMRI parameters (infarct size, ejection fraction, and microvascular obstruction), inflammatory biomarkers (high-sensitivity CRP and IL-6), and exploratory renal protective markers. Patients were stratified as positive or negative for CMV based on the serological status. For the assessment of effector memory T lymphocyte numbers blood monitoring followed on day 1 and 90 post STEMI.

Bulk TCR repertoire sequencing and analysis from cardiac tissue and blood patient samples

Bulk TCRβ repertoire analysis was performed on the two independent datasets, the blood samples from the ETiCS in Würzburg and the post-mortem cardiac tissue from Graz. TCR repertoire sequencing from myocardial tissue was performed at the Institute of Pathology at the Medical University of Graz. DNA/RNA was extracted from 3-5 10µM thick sections cut from FFPE tissue blocks using the Maxwell 16 FFPE Tissue DNA or RNA Purification Kit according to manufacturers instructions. Nucleic acids were eluted in a final volume of 45µl DNAse free water. Extracted DNA was subjected to multiplexed PCR using primers for the TCRB locus (26297338) or the TCRG locus (LymphoTrack TRG Assay - PGM, Invivoscribe). Additionally, amplicons were generated from cDNA using primers binding to V and J Regions of the TCR. PCR amplicons were end-repaired, adapter ligated and post amplified using the Ion Xpress Plus Fragment Library Kit (Thermo Fisher Scientific). Amplicons were sequenced to sufficient depth on the Ion Torrent S5 instrument using the 530 chip kit and 400bp chemistry. Raw data was quality checked and demultiplexed (FastQ data) and analysed with IMonitor script, custom perl/python scripts and/or proprietary software supplied by Invivoscribe (Lymphotrack DX Software). Briefly, amplicons were filtered to exclude truncated and artefactual sequences and mapped to TCRB/G references. For all samples (FFPE and blood), a list of normalized TRBV-CDR3-TRBJ and TRAV-CDR3-TRAJ sequences was obtained and stored in the i3 private secured TCR database for further analysis.

MiXCR output files from both datasets (Graz and Würzburg) were processed using the immunarch R package (version 1.0.0). Productive CDR3 sequences were extracted using the repLoad() function. For the ETiCS data set, only CDR3β sequences were retained, and cutoffs of at least 50 clonal counts and clonal proportions between 0.01% and 20% were selected from each sample for downstream analysis. Owing to the scarcity of T cells and TCR abundance from cardiac tissue samples, only CDR3β were sequenced, and cutoffs of at least 10 clonal counts and clonal proportions between 0.01% and 50% were selected. Experimentally validated human CMV-reactive or EBV-reactive TCRβ sequences were obtained from the McPAS-TCR database[31]. Metadata regarding the reactivity of the extracted TCRs to immunodominant CMV epitopes (pp65, CMV-NV9, IE-1, pp50, and UL29/28) and EBV epitopes (EBNA-6, EBNA-3A, BZLF-1, BMLF-1) was curated. Two compiled datasets of either ETiCS good and poor healer TCRs and CMV-curated TCRs or ETiCS good and poor healer TCRs, cardiac tissue TCRs and CMV-curated TCRs were generated. The same comparisons were performed with EBV-curated TCRs. Pairwise repertoire overlaps were computed using the Jaccard similarity index, comparing full-length CDR3β sequences between patient groups and the CMV or EBV reference. To account for both sequence sharing and abundance, custom R functions were implemented to compute weighted overlap scores. This included both the percentage of shared clonotypes (overlap frequency) and the average abundance of overlapping clones (percent clonal space), defined as the mean proportional space occupied by shared CDR3βs in each comparison. Overlaps were computed either

between patient groups (e.g., poor vs. good healers and CMV dataset) or between each patient group and epitope-specific subsets of the CMV TCR database. The results were visualized as dot plots where bubble size indicates overlap frequency and color represents percent clonal space. The tidyverse R package (version 2.0.0) was used for data wrangling, and ggplot2 (version 3.5.2) was used for visualization.

Histology

Hearts were fixed in 4% neutral buffered formalin and embedded in paraffin. Sections were cut at a thickness of 5 µm. Immunohistochemical staining for identification of infected cells was performed on paraffin sections using anti-IE1 antibody (clone IE1.01; Center for Proteomics, Faculty of Medicine, University of Rijeka, Rijeka, Croatia). Antibody binding was visualized with peroxidase-conjugated goat anti-mouse IgG antibody (Jackson ImmunoResearch Laboratory), followed with 3,39-diaminobenzidine as a chromogen (Dako). Slides were counterstained with Shandon hematoxylin and analyzed on DP 71 camera mounted on an BX 51 microscope (Olympus). In all experiments, liver tissue from MCMV-infected animals served as a positive control, while a heart from a non-infected animal was used as a negative control. The liver was selected as the positive control because it is extensively infected during acute infection, allowing positive cells to be clearly and reliably identified. In addition, an isotype control was included in all immunohistochemical stainings to confirm staining specificity. Picrosirius red staining was performed on paraffin sections and counterstained with Shadon hematoxylin. Fibrosis quantification was performed using a supervised pixel classification in ilastik (v1.4.0.post1). A pixel classification model was trained using the Random Forest classifier to assign each pixel to one of four classes: tissue, fibrosis, empty spaces, and scale bar. Training annotations were manually drawn on multiple representative images to capture variability in staining intensity, texture, and tissue morphology. The trained classifier was applied to all images to generate simple segmentation maps, in which each pixel was assigned a discrete class label. Segmentation images were processed using CellProfiler (v4.2.6). Binary masks were generated for fibrosis and for total tissue. The area occupied by fibrosis and by total tissue was measured on a per-image basis using the MeasureImageAreaOccupied module. Fibrosis burden was expressed as the percentage of fibrotic area relative to total tissue area. Representative images were selected after completion of the quantitative analysis and were chosen for the main and the supplemental figures to reflect the average phenotype observed within each respective experimental group.

Statistical analysis of mouse experiments

Data were analysed using GraphPad Prism 10.4.1 software. The data presenting the differences between two groups were analysed using two-tailed unpaired Student's *t*-tests, while comparison of more than two groups was assessed by using One-way ANOVA and performing Tukey's multiple comparisons test. Kolmogorov-Smirnov Test was used to assess normality. $P<0.05$ indicates statistical significance of the analysed sample compared to the controls.

Supplemental table 1: TotalSeq C antibodies used in sc-RNA sequencing.

Antibody	Sequencing runs/Experimental condition
TotalSeq™-C0301 anti-mouse Hashtag 1	seq run 1 MI, seq run 2 MCMV MI
TotalSeq™-C0302 anti-mouse Hashtag 2	seq run 1 MI, seq run 2 MI
TotalSeq™-C0303 anti-mouse Hashtag 3	seq run 1 MI, seq run 2 MI
TotalSeq™-C0304 anti-mouse Hashtag 4	seq run 1 MCMV MI, seq run 2 MI
TotalSeq™-C0305 anti-mouse Hashtag 5	seq run 1 MCMV MI, seq run 3 MI
TotalSeq™-C0306 anti-mouse Hashtag 6	seq run 1 MCMV MI, seq run 3 MI
TotalSeq™-C0307 anti-mouse Hashtag 7	seq run 1 MCMV MI, seq run 3 MCMV MI
TotalSeq™-C0308 anti-mouse Hashtag 8	seq run 1 MCMV MI, seq run 3 MCMV MI
TotalSeq™-C0309 anti-mouse Hashtag 9	seq run 3 MCMV MI
TotalSeq™-C0002 anti-mouse CD8a	seq run 1,2,3 all mice
TotalSeq™-C0001 anti-mouse CD4	seq run 1,2,3 all mice
TotalSeq™-C0987 anti-Allophycocyanin (APC)	seq run 1,2 all mice
TotalSeq™-C0436 anti-Biotin	seq run 1 all mice
TotalSeq™-C0911 anti-phycoerythrin (PE)	seq run 2,3 all mice

Supplemental table 2: Cardiac MRI parameters from the placebo group of FRACTAL trial[24]

	CytomegalovirusIgG	N	Mean	Std. Deviation	Std. Error Mean
LVEDV	positive	11	172,36	24,804	7,479
	negative	17	167,00	33,795	8,197
LVESV	positive	11	95,45	18,705	5,640
	negative	17	87,65	23,646	5,735
LVSV	positive	11	77,00	18,089	5,454
	negative	17	79,35	15,133	3,670
LVEF	positive	11	44,609	7,9236	2,3890
	negative	17	48,647	5,7205	1,3874

Supplemental table 3: Correlation parameters between infarct size of CMV-positive STEMI patients 3 days post STEMI, reperfusion and various CD8 T cell-parameters (left column) analyzed from the patients' blood samples. *. Correlation is significant at the 0.05 level (2-tailed). **. Correlation is significant at the 0.01 level (2-tailed).

		**GAD5_SD__perc_B**
**LVEF_(%)**	Pearson Correlation	-.564**
	Sig. (2-tailed)	0,003
	N	26
**Cd8 AbsTc**	Pearson Correlation	0,365
	Sig. (2-tailed)	0,066
	N	26
**Cd8 (%)**	Pearson Correlation	0,196
	Sig. (2-tailed)	0,336
	N	26
**Cd8 Tem (%)**	Pearson Correlation	-0,123
	Sig. (2-tailed)	0,551
	N	26
**Cd8 Temra (%)**	Pearson Correlation	0,380
	Sig. (2-tailed)	0,055
	N	26
**Cd8Cd28$^-$Cx3Cr1$^+$(%)**	Pearson Correlation	0,185
	Sig. (2-tailed)	0,365
	N	26
**Cd8Cd28$^-$Cx3Cr1$^+$ Abs.Nr.**	Pearson Correlation	0,347
	Sig. (2-tailed)	0,083
	N	26
**Cd8Cx3Cr1$^+$ Abs.Nr.**	Pearson Correlation	0,382
	Sig. (2-tailed)	0,054
	N	26
**Cd8Tem Abs.Nr.**	Pearson Correlation	0,097
	Sig. (2-tailed)	0,637
	N	26
**Cd8Temra Abs.Nr.**	Pearson Correlation	.435*
	Sig. (2-tailed)	0,026
	N	26

GAD: Gadolinium, LVEF: Left Ventricular Ejection Fraction, Tem: T effector memory cells, Temra: Terminal Effector Memory cells re-expressing CD45RA, Abs.Nr.: Absolute Numbers, AbsTc: Absolute T cells

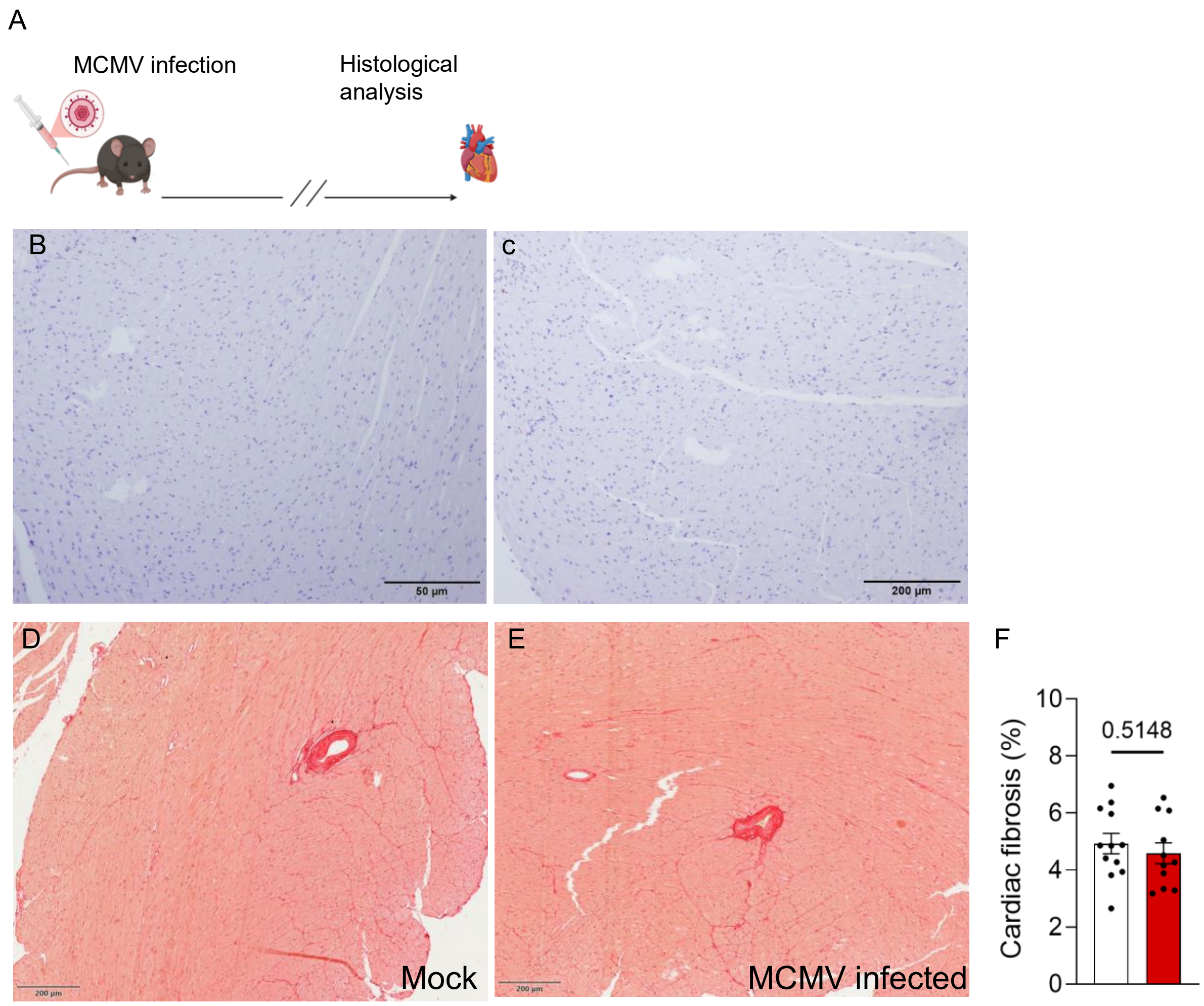

Supplemental Figure 1. A. Scheme. Mice were infected with 2x10^5 PFU of Δm157 MCMV. Non-infected animals were mock treated with the same volume of sterile PBS. **B, C.** Immunohistochemical IE-1 staining counterstained with hematoxylin showing no signs of overt cardiac pathology and tissue necrosis on day 5 after infection (n=4). **D, E .** Fibrosis staining with picrosirius red of mock infected controls (n=12) and MCMV infected mice (n=10) 12 months after infection. **F.** Quantification of percent fibrosis. Statistical analysis was performed using unpaired Student t-test.

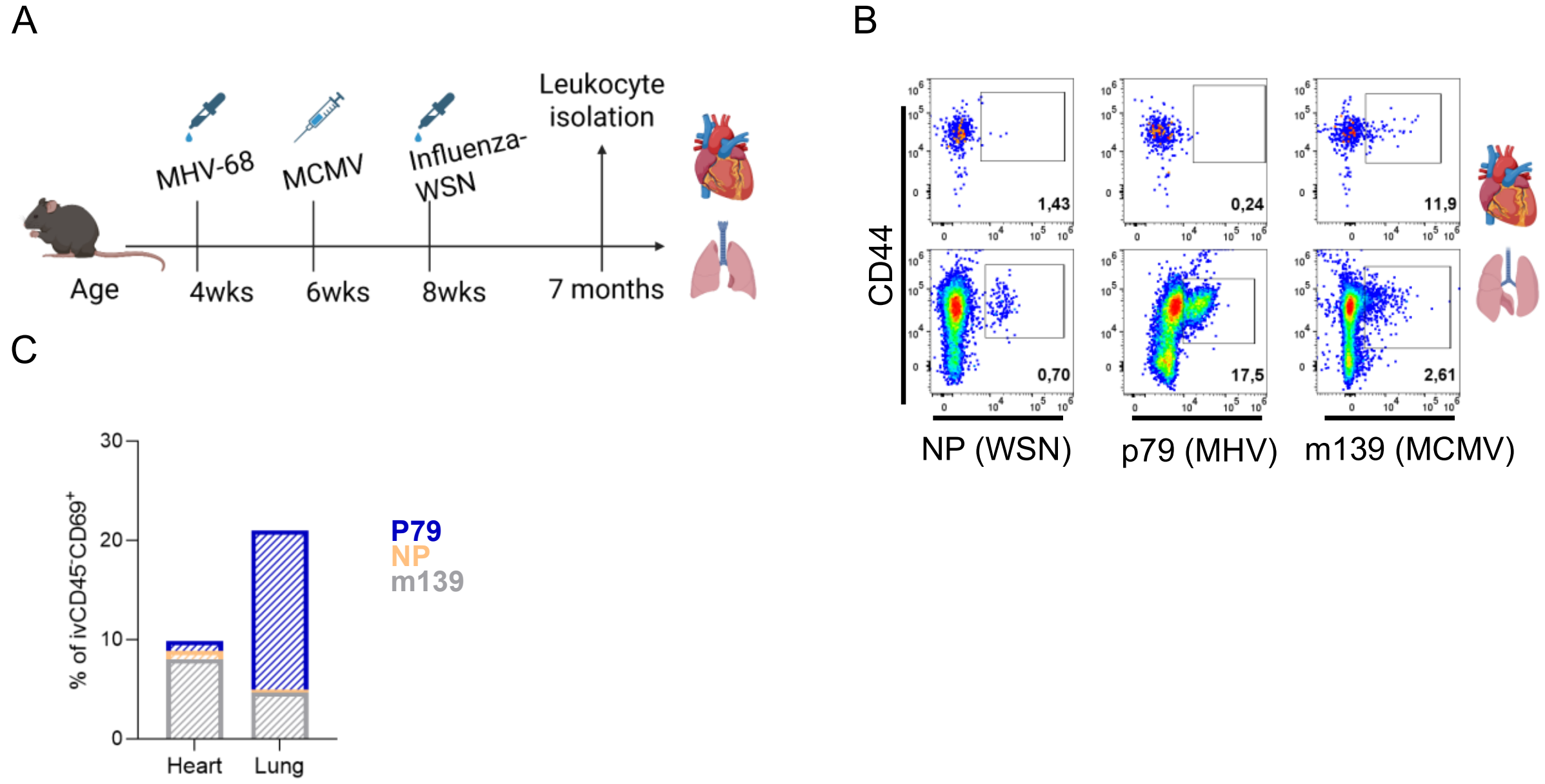

Supplementary figure 2. A. Sequential infection of mice with MHV-68 i.n., WSN i.n., MCMV i.p. and analysis of leukocytes from heart and lung at 7 months post last infection. **B.** Representative FACS plots of tissue residing (ivCD45^{-}) virus-specific CD8^{+} T cells as identified by tetramer staining in the hearts (upper row) and lungs (lower row). Intravenously injected CD45 was used to identify extravascular, tissue-residing CD8^{+} T cells. NP epitope-tetramer complex was used for influenza A/WSN, p79 MHC-I tetramer (Kb/ORF61$_{524–531}$ TSINFVKI) was used for MHV-68 and m139 for MCMV. **C.** Quantification of the percentage of virus-specific CD8^{+} T cells in the cardiac and pulmonary tissue (n=4/tissue)

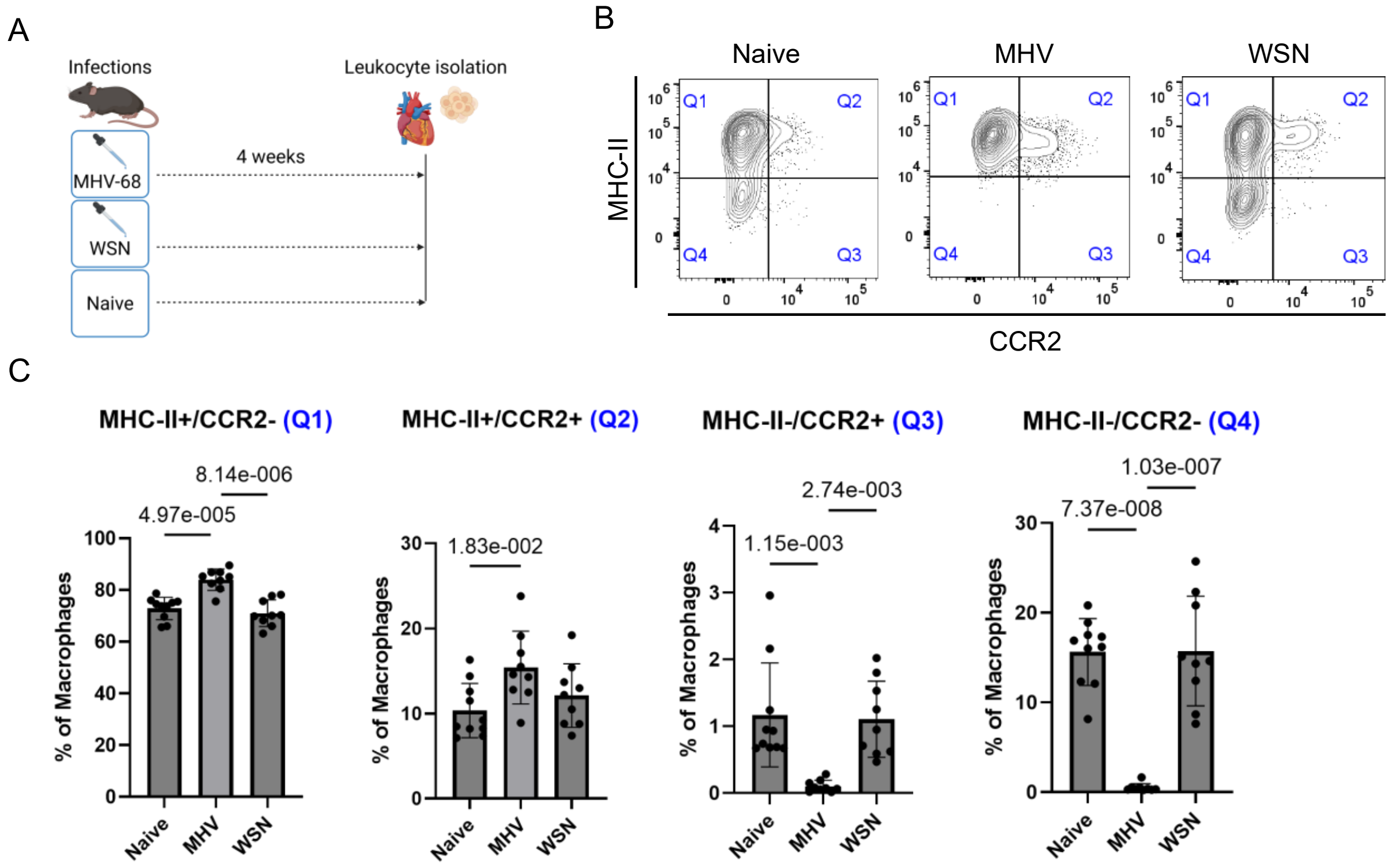

Supplementary figure 3. A. Leukocyte isolation from the heart of naïve mice and mice infected with MHV-68 or WSN individually. **B.** Representative FACS plots of tissue-residing (ivCD45^{-}) macrophages (pre-gated on CD64^{+}CD11b^{+} cells) and **C.** graphs of Q1-Q4 populations. Statistics were performed using one-way ANOVA, p value is numerically indicated, non-significant values are not shown. In the naïve n=10 and in the infected n=9.

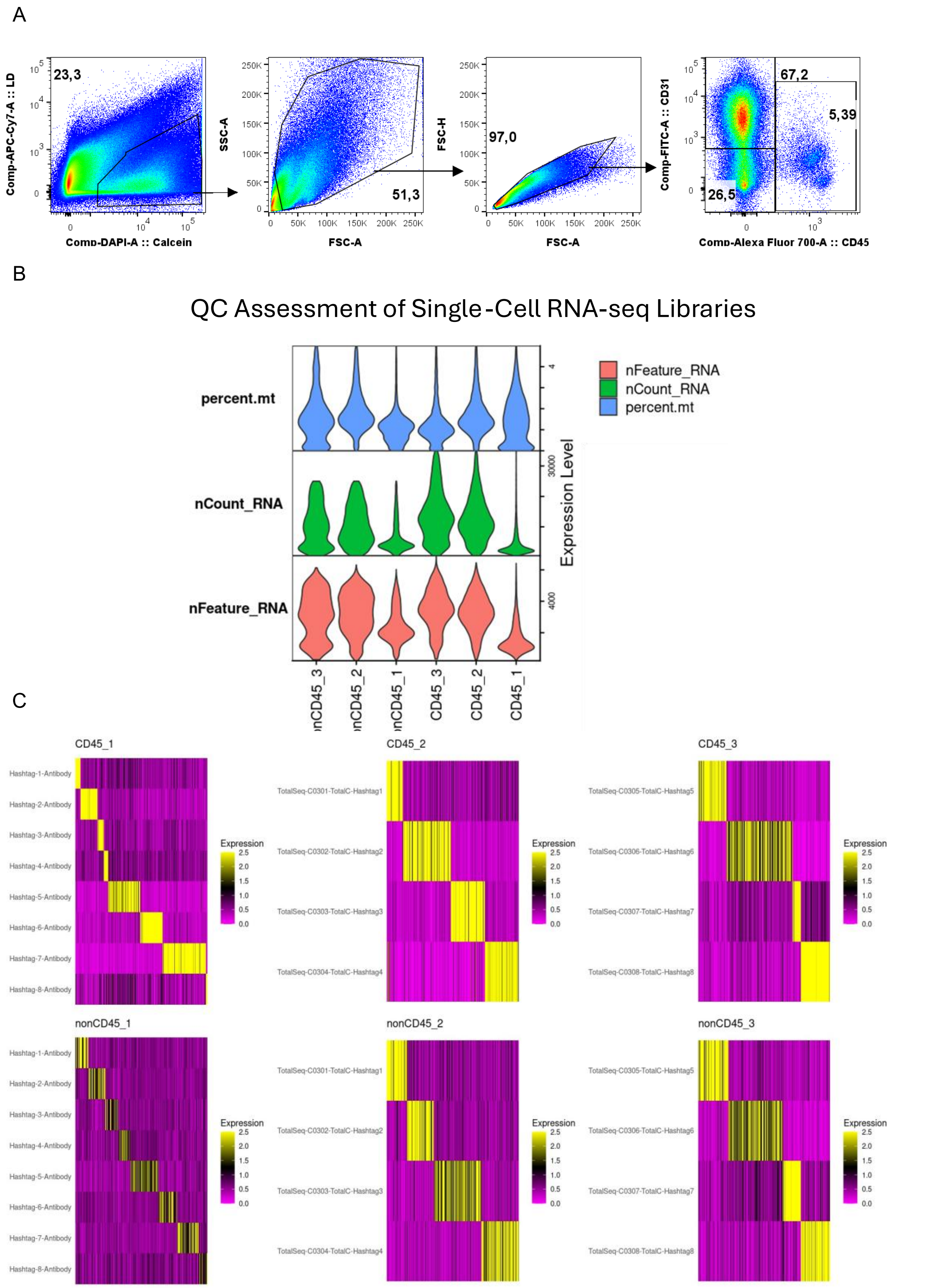

Supplementary figure 4. A. Sorting strategy for Calcein+ viable CD45+ CD31- immune cells, CD45- CD31+ endothelium, or CD45- CD31- mesenchymal cells. **B**. Violin plots depict the percentage of mitochondrial genes, the RNA count, and the number of genes detected, per cell from each single-cell library. **C**. Heatmaps indicating the barcoding strategy for demultiplexing individual samples that were pooled in each library.

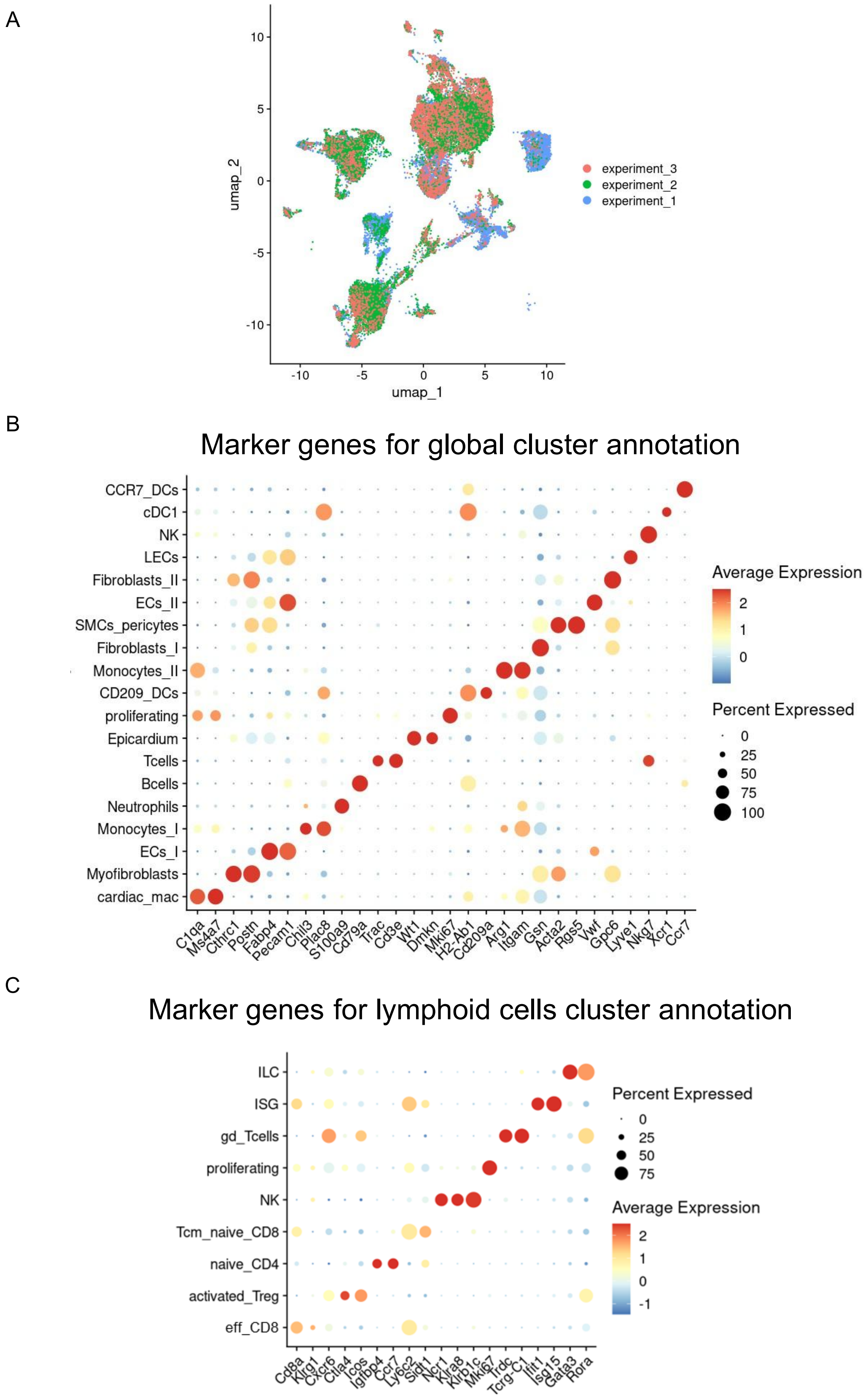

Supplementary figure 5. A. UMAP representation of the three individual sc-RNA sequencing experiments. **B**. Dot plot showing marker genes defining each cluster of cells from the global annotation of the sc-RNA sequencing. Size of the dot represents the percentage of cells expressing each gene within each population and color represents the expression level. Red indicating upregulation and blue indicates downregulation. **C**. Dot plot showing marker genes defining each cluster of lymphoid cells from the global annotation of the sc-RNA sequencing.

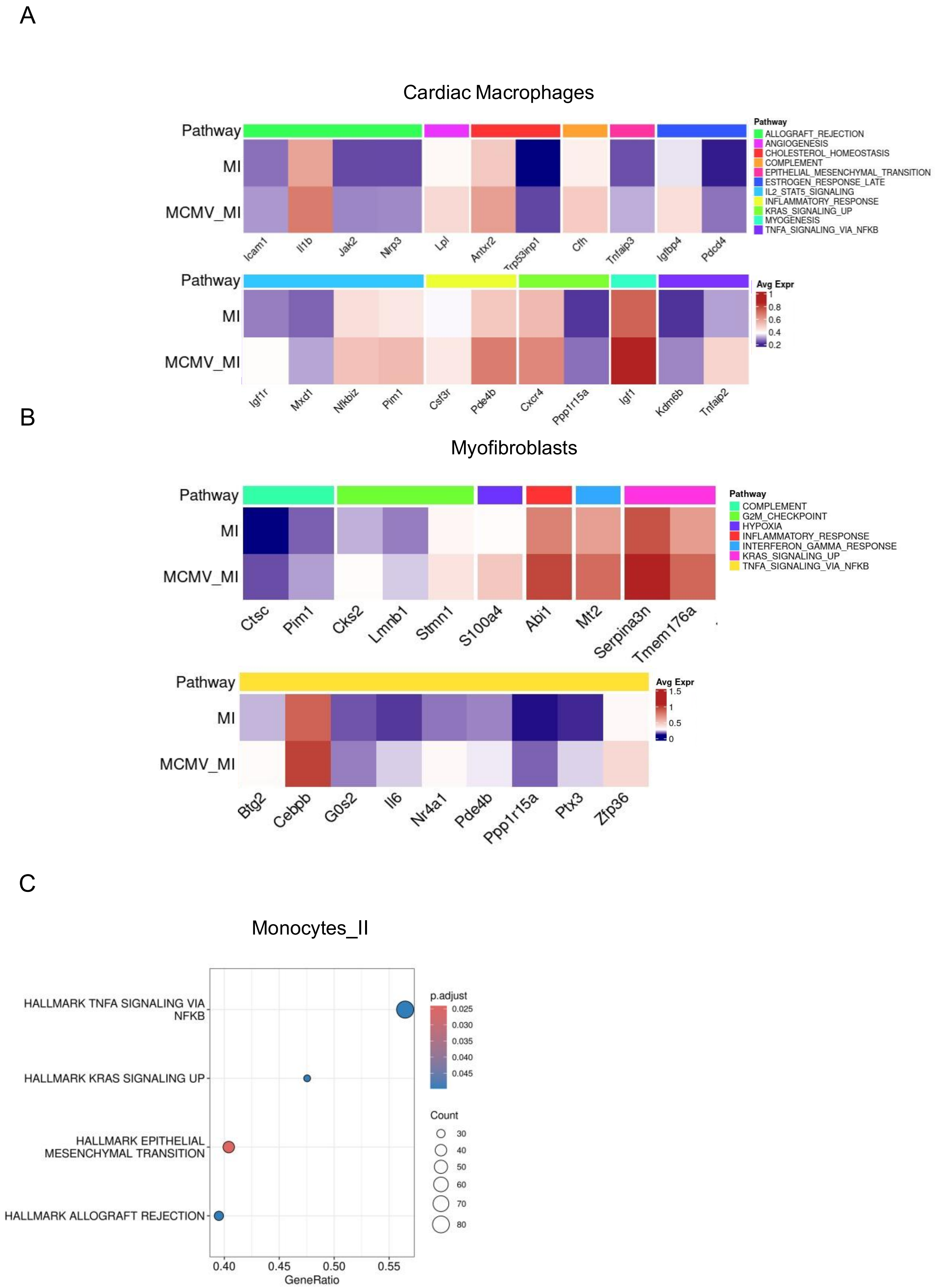

Supplementary figure 6. Heatmap of representative core genes from GSEA-enriched pathways in **A.** cardiac macrophages, and **B.** myofibroblasts. Average expression values are compared between MCMV+MI and MI conditions for each selected gene (up to four per pathway), grouped by pathways. Pathways are color-annotated above the columns. Only core enrichment genes with log2 fold-change > 0.4 and detected in >20% of cells in the upregulated

subset are included. **C.** Gene set enrichment analysis of the top upregulated genes from the Monocytes_II subset in MCMV+MI mice compared to MI, followed by heatmap of representative core genes from GSEA-enriched pathways. **d.** Network analysis, using the GLIPH2 algorithm, of motif enrichment in the top-expanded CDR3 sequences. Each node represents a unique CDR3 sequence that forms a cluster based on sharing a core CDR3 motif. Core motifs are restricted to the region with high contact probability with an antigen presented on an MHC molecule[32]. A reference database of 78,116 murine CDR3 sequences from naive TCR repertoire was used to control for random motif enrichment. Node color indicates the source of the TCR CDR3 sequence: purple is MCMV + MI, yellow is MI, and orange is McPAS-TCR database[31].

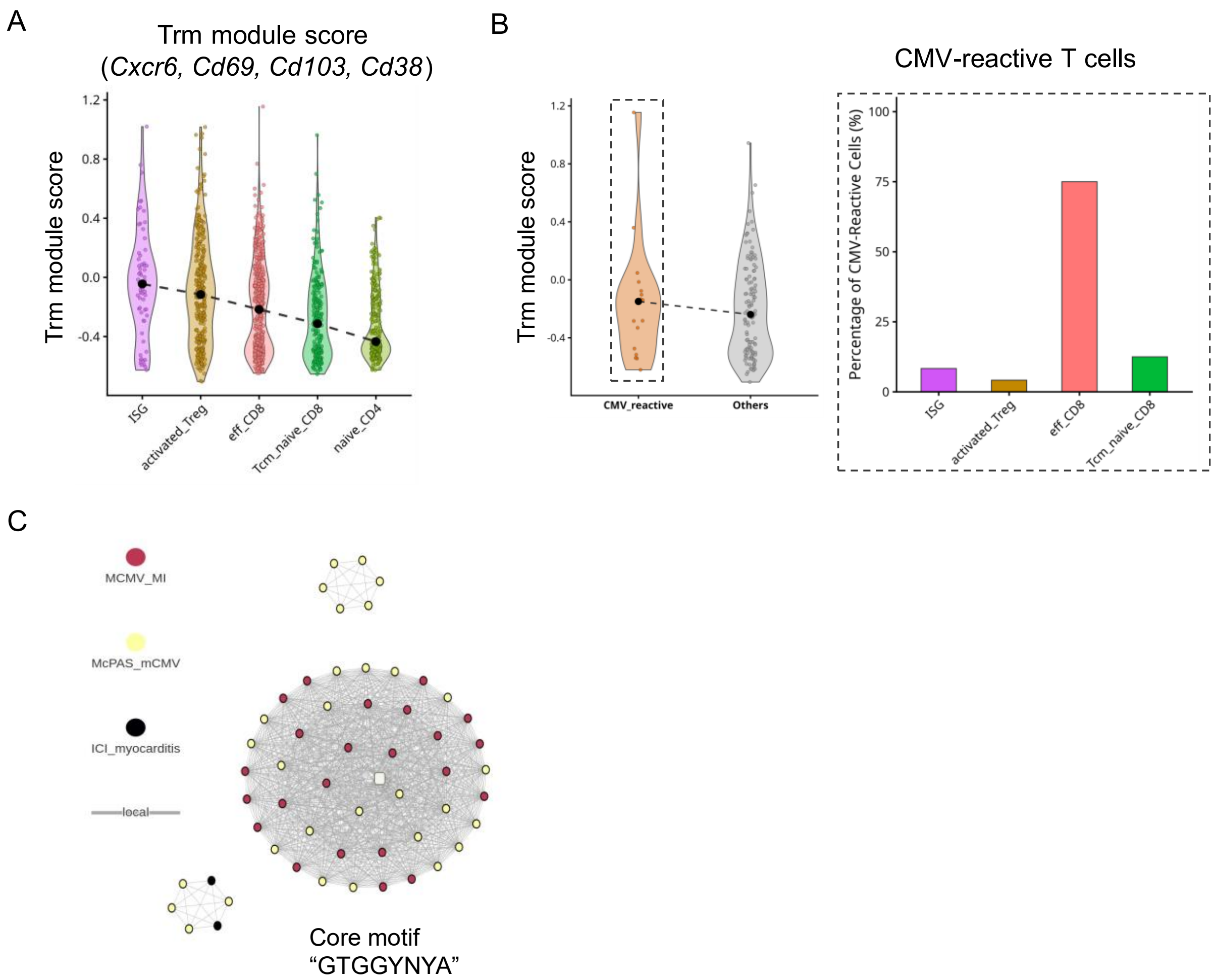

Supplementary figure 7. A. Violin plot of a compiled tissue residency genes module score (*Cxcr6, Cd69, Cd103, Cd38*) across T cell subsets infiltrating the infarcted myocardial tissue. **B.** (Left) Violin plot of the tissue residency module score enrichment in T cells with annotated TCRs that show CMV-reactivity compared to non-CMV annotated TCRs. (Right) Barplot of T cell phenotype distribution across the T cells with annotated TCRs that show CMV-reactivity. **C.** Network analysis, using the GLIPH2 algorithm, of motif enrichment in the top-expanded CDR3 sequences. Each node represents a unique CDR3 sequence that forms a cluster based on sharing a core CDR3 motif. Core motifs are restricted to the region with high contact probability with an antigen presented on an MHC molecule[17]. A reference database of 78,116 murine CDR3 sequences from naive TCR repertoire was used to control for random motif enrichment. Node color indicates the source of the TCR CDR3 sequence: purple is MCMV + MI, yellow is MI, and orange is McPAS-TCR database[19].

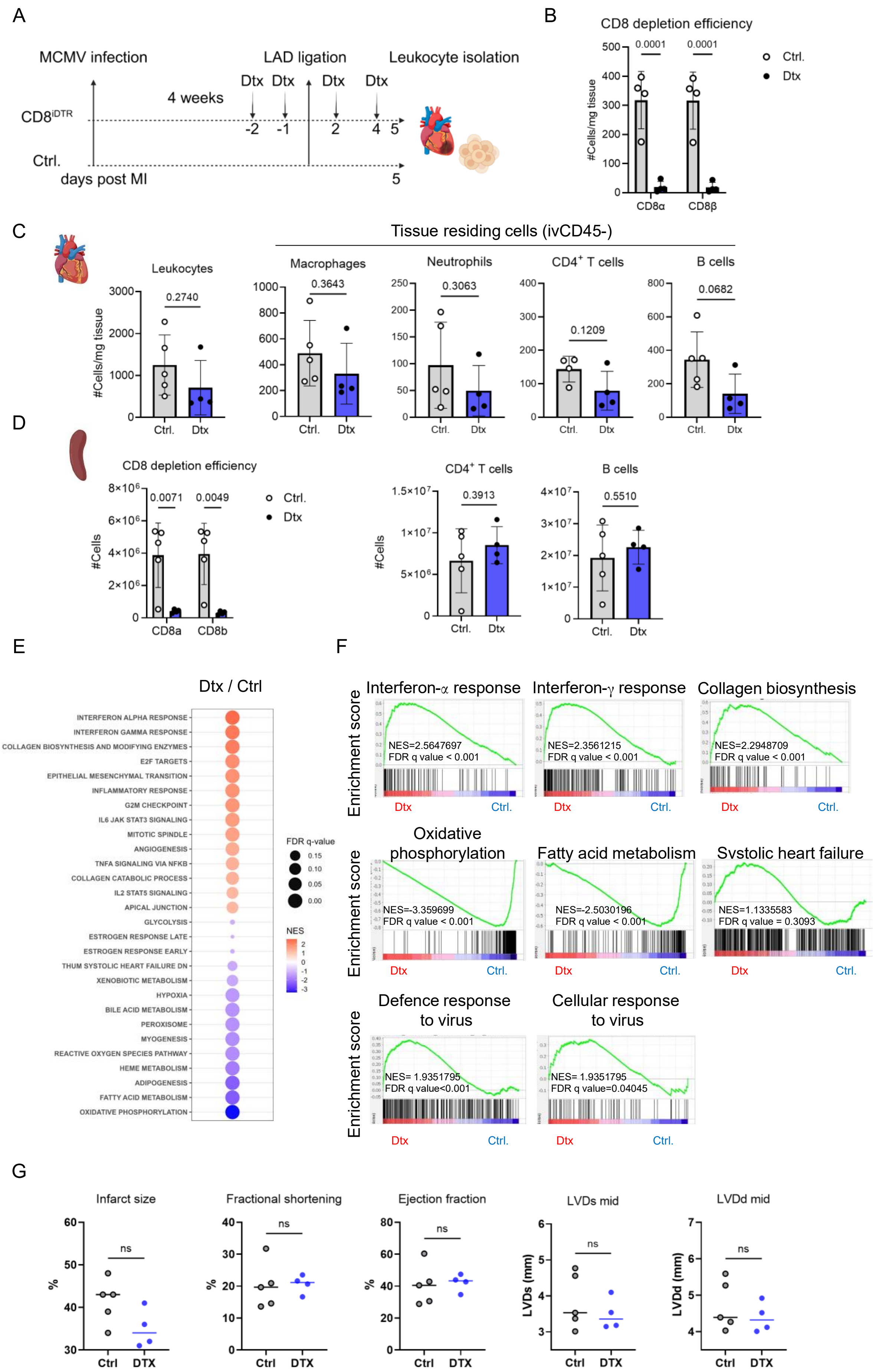
A
MCMV infection
LAD ligation
Leukocyte isolation
4 weeks
Dtx Dtx Dtx Dtx
CD8iDTR
-2 -1 2 4 5
Ctrl.
days post MI
5
B
CD8 depletion efficiency
0.0001
0.0001
Ctrl.
Dtx
#Cells/mg tissue
CD8α
CD8β
C
Tissue residing cells (ivCD45-)
Leukocytes
0.2740
Macrophages
0.3643
Neutrophils
0.3063
CD4+ T cells
0.1209
B cells
0.0682
#Cells/mg tissue
Ctrl.
Dtx
D
CD8 depletion efficiency
0.0071
0.0049
Ctrl.
Dtx
#Cells
CD8a
CD8b
CD4+ T cells
0.3913
B cells
0.5510
E
Dtx / Ctrl
INTERFERON ALPHA RESPONSE
INTERFERON GAMMA RESPONSE
COLLAGEN BIOSYNTHESIS AND MODIFYING ENZYMES
E2F TARGETS
EPITHELIAL MESENCHYMAL TRANSITION
INFLAMMATORY RESPONSE
G2M CHECKPOINT
IL6 JAK STAT3 SIGNALING
MITOTIC SPINDLE
ANGIOGENESIS
TNFA SIGNALING VIA NFKB
COLLAGEN CATABOLIC PROCESS
IL2 STAT5 SIGNALING
APICAL JUNCTION
GLYCOLYSIS
ESTROGEN RESPONSE LATE
ESTROGEN RESPONSE EARLY
THUM SYSTOLIC HEART FAILURE DN
XENOBIOTIC METABOLISM
HYPOXIA
BILE ACID METABOLISM
PEROXISOME
MYOGENESIS
REACTIVE OXYGEN SPECIES PATHWAY
HEME METABOLISM
ADIPOGENESIS
FATTY ACID METABOLISM
OXIDATIVE PHOSPHORYLATION
FDR q-value
0.15
0.10
0.05
0.00
NES
F
Interferon-α response
NES=2.5647697
FDR q value < 0.001
Interferon-γ response
NES=2.3561215
FDR q value < 0.001
Collagen biosynthesis
NES=2.2948709
FDR q value < 0.001
Oxidative phosphorylation
NES=-3.359699
FDR q value < 0.001
Fatty acid metabolism
NES=-2.5030196
FDR q value < 0.001
Systolic heart failure
NES=1.1335583
FDR q value = 0.3093
Defence response to virus
NES= 1.9351795
FDR q value<0.001
Cellular response to virus
NES= 1.9351795
FDR q value=0.04045
Enrichment score
Dtx
Ctrl.
G
Infarct size
Fractional shortening
Ejection fraction
LVDs mid
LVDd mid
ns
%
LVDs (mm)
LVDd (mm)
Ctrl
DTX

Supplementary figure 8. A. Scheme of $CD8^+$ T cell depletion. Four- to seven-week-old male $CD8a^{iDTR}$ mice were infected i.p. with MCMV and subjected to MI 4 weeks later. Diphtheria toxin (Dtx) was administered on days -2, -1, 2, and 4 relative to MI, control mice received no Dtx. Mice were sacrificed on day 5 after MI. **B.** CD8 T cell depletion efficiency illustrated as number of CD8α and CD8β cells/mg heart tissue 5 days post MI. **C.** Number of cells/mg heart tissue calculated for leukocytes and tissue residing ($ivCD45^-$) macrophages ($CD64^+$), Neutrophils ($Ly6G^+$), $CD4^+$ and B cells. **D.** CD8 depletion efficiency in the spleen and number of total cells calculated for $CD4^+$ and B cells. **E.** Dot-plot of significant gene set up- and down-regulation as obtained from bulk RNA sequencing data of CD8-depleted and Ctrl. hearts. Color scale represents the normalized enrichment score (NES), and circle size shows the false discovery rate (FDR). **F.** Gene set enrichment analysis of Interferon-α and -γ response, collagen biosynthesis, oxidative phosphorylation, fatty acid metabolism, systolic heart failure, defence and cellular response to virus among the two groups. Blue indicates transcript downregulation while red indicates upregulation. Data shown from one experiment with n=4 mice/condition (Dtx and Ctrl.) and mice included if infarct size >30%. (**B, C, D**) For the Ctrl. n=5 and for the Dtx n=4. **G.** Infarct size, fractional shortening and ejection fraction measured of the left ventricle at the mid-papillary level, just above the akinetic area induced by MI, as well as diastolic and systolic inner diameters of the left ventricle. In Ctrl. n=5 and in DTX n=4.

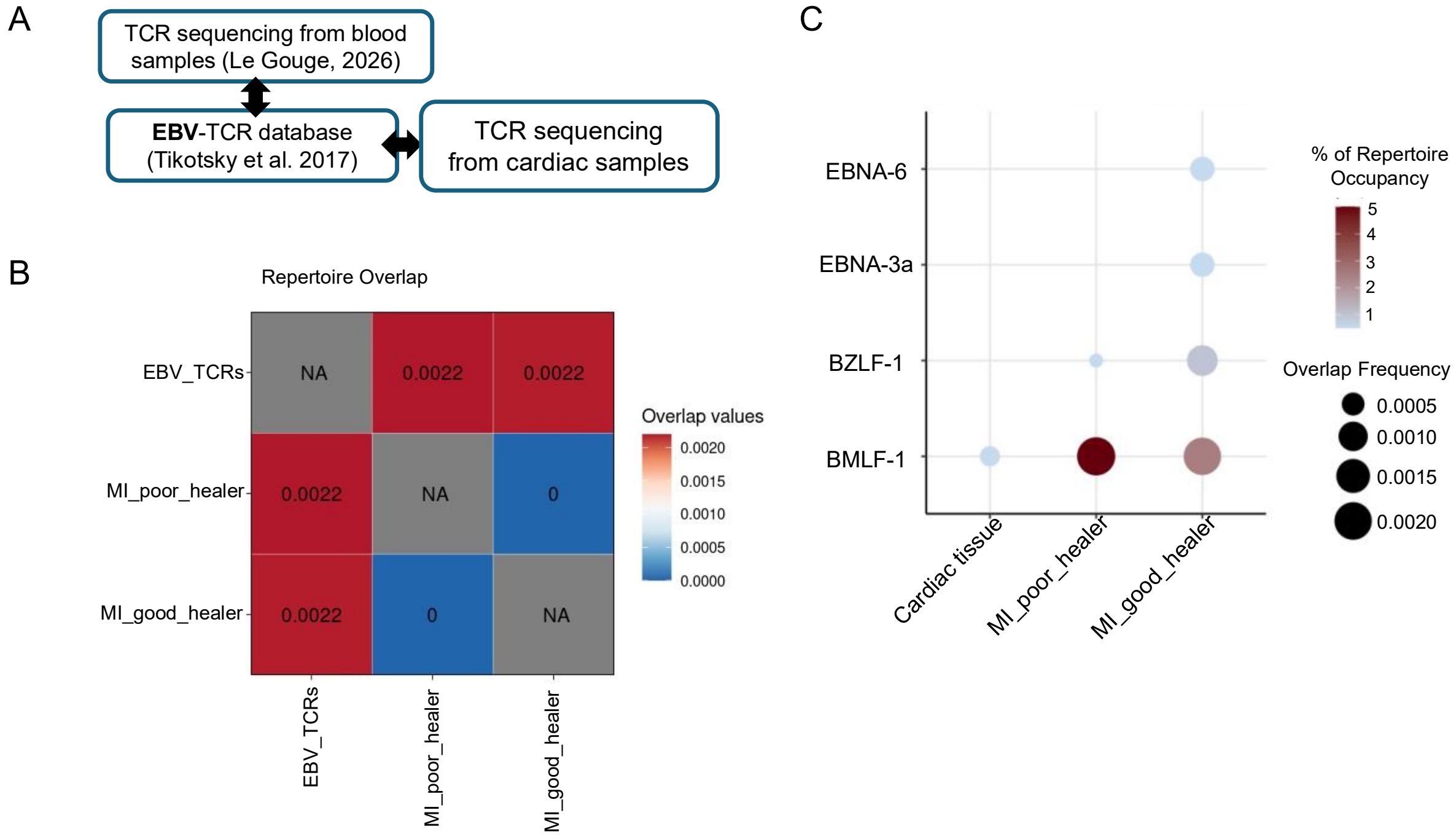

Supplementary figure 9. A. TCR sequencing from cardiac autopsies of acute-MI samples integrated with TCRs from blood samples of the ETiCS cohort, and EBV-annotated TCRs[31]. **B.** Heatmap of Jaccard similarity index based on exact CDR3b sequence matches between EBV-annotated TCRs and TCR repertoires from blood samples of poor or good post-MI healing groups. Color intensity reflects the degree of clonal similarity, with red indicating higher overlap. **C.** Dotplot of Jaccard similarity index between TCR repertoires from blood samples of poor or good healers, and from cardiac autopsies comparing their specificity to defined EBV epitopes. Dot size reflects the degree of clonal similarity, and color intensity indicates the degree of clonal expansion of EBV TCRs within each analyzed group.
